# Acknowledgment to the Reviewers of *Diagnostics* in 2022

**DOI:** 10.3390/diagnostics13030353

**Published:** 2023-01-18

**Authors:** Diagnostics Editorial Office

**Affiliations:** MDPI AG, St. Alban-Anlage 66, 4052 Basel, Switzerland

High-quality academic publishing is built on rigorous peer review. *Diagnostics* was able to uphold its high standards for published papers due to the outstanding efforts of our reviewers. Thanks to the efforts of our reviewers in 2022, the median time to first decision was 18 days and the median time to publication was 38 days. Regardless of whether the articles they examined were ultimately published, the editors would like to express their appreciation and thank the following reviewers for the time and dedication that they have shown *Diagnostics*:
Aalap ChokshiKrzysztof SzyfterAaron FensterKuldeep GuptaAaron HeffernanKumiko SaekiAaron PollettKun QinAatke Van Der MaasKun YangAbbas F. AlmullaKunal AjmeraAbdelhameed IbrahimKundi YangAbdelmoneim SuliemanKun-Eek KilAbdelmotagaly ElgaladKun-Lin TsaiAbdol PaghehKuo-Hsuan HungAbdolhassan KazemiKuo-Hu ChenAbdolkarim HosseiniKurt A. JellingerAbdolreza JamilianKurt SvärdsuddAbdul NoorKwang-sig LeeAbdul SirajKwok Kan SoAbdülkadir TunçKyong ChoAbdulkarim HasanKyu Young ChoiAbdulkarim Jafar KarimKyung Eun LeeAbdullah AlanaziKyung Jin SeoAbdullah BayramKyunghoon MinAbdullah Bin ShamsKyung-Hun KimAbdullah DemirtaşKyung-Yong ChungAbdullah TariqueKyuseok KimAbedalrhman AlkhateebLadislav DokládalAbhaya Chandra DasLaetitia ChauviereAbhijit MondalLaís Canniatti BrazacaAbhijit PakhareLaith AbualigahAbhinay GontuLaith AlzubaidiAbhishek KumarLaith SultanAbhishek MishraLakshmanakumar KinthadaAbraham PouliakisLamia Ait-AliAda ColluraLampros PapadimitriouAdam GlowaczLana NežićAdam GołąbLarisa AnghelAdam J. JanasLarisa LitvinovaAdam ReichLarisa PinteAdam WittekLarisa Viktorovna SuturinaAdeboye AzeezLars Birger LönnAdel FateasLars KattnerAdel S. AlaglLars NorgrenAdela Enache-AngoulvantLassina BarroAdelaida AvinoLászló PohlAdem OzcelikLaszlo TabarAdi BeharLatika GuptaAdil Mehmood KhanLaura BurattiniAditya BansalLaura C. Hernández-RamírezAditya GanjuLaura De PlanoAdnan KhanLaura Giuseppina Di PasquaAdolfo RomeroLaura MarchiAdriaan Erasmus BassonLaura MaziluAdrian Elmi-TeranderLaura PellegrinelliAdrian GillissenLaura PetrarcaAdrian GrozaLaura SalvadoriAdrian Kah Heng ChiowLaura SebastianiAdrian LitaLaura VicenteAdrian NeaguLaura VitielloAdrian WlodarczakLaura-Cristina RusuAdriana GrigorașLaurel O. SillerudAdriano PintoLaurence RozenAdrienne L. GrzendaLaurent BélecAdrija HajraLaurent MetzingerAfshin AkhshaniLaurent MullerAfshin ShoeibiLaurent PeyrodieAftab AlamLaurent SuppanAftab Aslam Parwaz KhanLaurentia Nicoleta GaleșAgata Poniewierska-BaranLaurentiu PirteaAgata RaniszewskaLaurentiu PopAgata StanekLaurie M. ElitAglaja StirnLea AzourÁgnes CsivincsikLeal G. SeguraAgnes SchröderLeandro Dias Da SilvaAgnese SbrolliniLeandro SantiagoAgnieszka A. KołodzińskaLech B. DobrzańskiAgnieszka AdamczukLechuang ChenAgnieszka GerkowiczLee Andrew GoeddelAgnieszka Jagiello-GruszfeldLee Samüel WannAgnieszka KamińskaLei YeAgnieszka KordekLei ZhouAgnieszka Łupicka-SłowikLeif BülowAgnieszka ŚmieszekLenka VysloužilováAgnieszka StawarskaLeny JoseAgustin CastiellaLeo KevinAgustín Díaz-ÁlvarezLeo MassariAh Young JungLeona GilbertAharon WegnerLeonardo BascoAhmad AfifyLeonardo CentonzeAhmad AlbaghdadiLeonid Pavlovich ChurilovAhmad MohammadLeopold EckhartAhmed AlshareefLeslie Chavez-GalanAhmed ElkamhawyLeslie Marisol Lugo GavidiaAhmed ElmassryLetizia Li PianiAhmed Hassan Hemdan MohamedLev KazakovtsevAhmed KhaledLeyre ZubiriAhmed Mohamed Abdel-AzeemLi ZuoAhmed S. MandourLia Mara DiţuAhmed SedikLi-An ChuAhmed Shihab AlbahriLiana PlesAhmet AltanLiana TodorAhsan JavedLiang Ting LinAi Ling HourLiang-In LinAida PetcaLiangqun LuAidel SalihLiang-Tseng KuoAïmen KhacharemLiangyi ChenAirlane Pereira AlencarLianhua DongAi-Ru HsiehLianyong QiAjay KevatLi-Ching ChangAjeet KaushikLicun WuAjinkya PawarLiepa BikulčienėAjoe John KattoorLijun YuanAjoy Prasad ShettyLikwang ChenAkanksha SharmaLilach SoreqAkari Nakamura-UtsunomiyaLin ZhaoAkhtar AkhtarLina BaranauskieneAkif TurnaLina DuAkifumi EnomotoLina PezzutiAkihide KondoLindsay HinckAkihiko HiyamaLing KuoAkiko HanyudaLing TangAkiko YokoiLing YinAkio NakashimaLingwen DingAkio ShimizuLinhua WangAkira SatohLiping ZhangAkira SugawaraLiqiang XiAkira YamamiyaLisa F. ShubitzAkshay GoelLisa J. UnderlandAkshay PandeyLisandro LovisoloAlaa Hazem GaafarLivia CarrascalAlain AttarLivia RuffiniAlain SarauxLiviu SteierAlan HaynesLixin RuiAlan P. SawchukLjubica ZupunskiAlanna StrongLjubiša Šaric̈Alba CorellLondon Lucien Peng Jin OoiAlban Fouasson-ChaillouxLong QianAlbert Estrugo-DevesaLongjiang ZhangAlbert FlotatsLopez GianlucaAlbert Thomas AnastasioLoredana MarcuAlberto Adanero VelascoLoredana NicolettiAlberto ChighineLoredana Sabina Cornelia ManolescuAlberto FerrareseLorena Elena MeliţAlberto Lo GulloLorena ZentilinAlberto MiceliLorenzo CeppiAlberto Molares VilaLorenzo CereserAlberto PalazzuoliLorenzo Diaz BalzaniAlberto ZamòLorenzo FaggioniAlceu De Souza BrittoLorenzo FranceschettiAldesia ProvenzanoLorenzo LippiAldo ClericoLorenzo LoffredoAldo NicosiaLorenzo MemeoAldona KasprzakLorenzo PaceAleix SolanesLorenzo UggaAlejandro Lucas BorjaLorenzo VassalloAlejandro Martín-GorgojoLory Saveria CrocèAlejandro Roldán-AlzateLouis ArnouldAlejandro SerrabloLouis TerriouAlejandro VallejoLouise PittAleksandar RakićLu WangAleksander ZwierzLubomír VojtíšekAleksandr PoliakovLubov TimchenkoAleksandra AsaturovaLuca AmbrosioAleksandra JungLuca ArcariAleksandra Piechota-PolanczykLuca ArdigòAleksandra S. KristoLuca BrazziAleksandra StupakLuca BurroniAleksandra SzymczakLuca CamoniAleksandras VilionskisLuca CevolaniAleksey Michailovich ChaulinLuca Di GianfrancescoAleš DvořákLuca EspositoAleš FidlerLuca FalzoneAlessandra AlciatiLuca FilippiAlessandra BorchielliniLuca FranchinAlessandra CancellieriLuca GallelliAlessandra PierangeliLuca GiacomelliAlessandra SorienteLuca Giovanni CampanaAlessandra ZilliLuca MagistrelliAlessandro Alessandro PalumboLuca MarsiliAlessandro ArenaLuca MenichettiAlessandro BeghiniLuca NeriAlessandro CapucciLuca NicosiaAlessandro CeliLuca Oscar Redaelli De ZinisAlessandro ComandoneLuca PalazzoloAlessandro De SireLuca RevelliAlessandro FascianiLuca SimulaAlessandro FranchiLuca SoraciAlessandro GabrielliLuca ValerioAlessandro GalaniLucas WesselAlessandro GambellaLucia MuracaAlessandro GranitoLucia StanciakovaAlessandro MalobertiLucia StančiakováAlessandro ManconLuciana CaenazzoAlessandro Maria SelvitellaLuciana VintiAlessandro MassaroLuciano PironeAlessandro OttaianoLucija BrezočnikAlessandro PerrellaLucinda BessaAlessandro PiniLucy A. CouplandAlessandro PosaLucy NorrisAlessandro RapisardaLudmila Danilowicz-SzymanowiczAlessandro RizzoLudmila LozneanuAlessandro ScalaLudwig WagnerAlessandro StefanoLue SunAlessia CarboniLuigi BennardoAlessia Maria CossuLuigi Della CorteAlessia RocchiLuigi Di TommasoAlessio Danilo ComisLuigi Federico RinaldiAlexander BerezinLuigi FortunaAlexander E. KabakovLuigi Francesco IannoneAlexander KleinLuigi GianturcoAlexander RufLuigi IraceAlexander SboevLuigi LoscoAlexander TamalunasLuigi MeccarielloAlexander V. ProninLuigi SantacroceAlexander Van SpeybroeckLuigi SchipsAlexandra BurluiLuigi TroisiAlexandra NikakiLuigia D. De FalcoAlexandra Peregrina LavínLuis Alberto Gaitán-CepedaAlexandra RoudenkoLuis CoelhoAlexandros DiamantisLuis Enrique CucaAlexandros LaiosLuis González-BayónAlexandros PatrianakosLuis JunqueraAlexandru Bogdan CiubaraLuis LarreaAlexandru BucurLuis Mariano EstebanAlexandru BurlacuLuís Pedro Ferreira RatoAlexandru CorlateanuLuis RamírezAlexandru EniuLuis RodrigoAlexandru Florin RogobeteLuisa PilanAlexandru PopaLuiz Carlos Conti De FreitasAlexandru VlasaLukas B. MoserAlexey ChubarovLukas LambertAlexey DeykinLukáš ŠkoloudíkAlexey SokolovLukasz KozlowskiAlexey SuminLukasz MalekAlexey SurovŁukasz SzeleszczukAlexey V. RakovŁukasz SzycAlexey ZaikinLuminita MoraruAlfizah HanafiahLung-Kwag PanAlfonso Maria PonsiglioneLut TamamAlfonso Martínez-NovaLu-Ting KuoAlfonso MastropietroLuyao ZhouAlfonso Salgado-AguayoLuz Kelly AnzolaAlfred O. AnkrahLuz MoranAlfredo CaturanoLydia Gimenez LlortAlfredo R. GalassiLydia Min-Ying SuAlfredo VellidoLyubov E. SalnikovaAli Abbasian ArdakaniMaarten Van CleeffAli AkalinMaarten W. NijkampAli Al KaissiMacarena Gomez-LiraAli Al-AhmadMacchi AlbertoAli BoolaniMaciej DabrowskiAli Mohammad AlqudahMaciej GawęckiAli Muhamed AliMaciej Iek SkrzypczakAli SelamatMaciej JozwikAli Shariq ImranMaciej MilanowskiAli Yavuz KarahanMaciej PolakAlice BonomiMaciej SosnowskiAlice VerticchioMaciej WalędziakAlicia Georgina Siordia-ReyesMaciej WiatrAlicia Rodríguez-BarberoMadhup RastogiAlicia Rodriguez-PlaMads Radmer JensenAlicia Saz-LaraMaenia ScarpinoAlin Laurenţiu TatuMagda ZanelliAlina ConstantinMagdalena Dutsch-WicherekAlisha PrasadMagdalena HagovskaAlka ChaubeyMagdalena KostkiewiczAllah Bux SarganoMagdalena LinkeAllison LewisMagdalena SkarzynskaAlmudena Alonso-OjembarrenaMagnus Bjorkavoll-BergsethAlok KumarMahdieh AlipourAlphus Dan WilsonMahmood BaraniAlpo VuorioMahmood Maher SalihAlteri ClaudiaMahmoud AhmedÁlvaro Francisco Lopes De SousaMahmoud ElbattahÁlvaro SobrinhoMahmoud El-MaghrabeyAlvise SernicolaMahmoud HassaballahAmad ZafarMahmoud HusseinAmal K. MitraMahmoud KandeelAmanda BrooksMahmud HasanAmanda LosiMahsa DabaghAmani Yousef OwdaMahshid NaghashpourAmaniel KefleyesusMaik KschischoAmany MohamedMaja PakižAmany SarhanMaja Rogić VidakovićAmarish YadavMaja SurbatovicAmber L. PondMajid JadidiAmbrogio P. LonderoMajid NiazkarAmeesh M. IsathMaju KuriakoseAmelia CasamassimiMakoto EmotoAmelia Franke-RadowieckaMakoto EndoAmelia Maria GamanMakoto HaradaAmeth Hawkins-VillarrealMakoto HasegawaAmeya P. NayateMalek MakkiAmir AbdoliMalene PedersenAmir FaisalMalgorzata KolodziejAmir H. GoldanMałgorzata Kotula-BalakAmir RavandiMałgorzata Plechawska-WójcikAmir ShemiraniMałgorzata Polz-DacewiczAmir SohrabiMałgorzata RogalińskaAmir-Houshang ShemiraniMałgorzata SzołkowskaAmirmasoud AhmadiMalgorzata Trofimiuk-MüldnerAmit AgrawalMałgorzata ZadurskaAmit GoelMałgorzata Zalewska-AdamiecAmit KumarMalik AydinAmit SinghMallesh KurakulaAmjad RehmanMalvinder S. ParmarAmmar EbrahimiMamoru AyusawaAmmar MohammedManabu KatoAmos M. YinnonManabu YamazakiAmr Mohamed Samy MahdyManal FawzyAmy L. ShafrirManal KleibAmyn M. RojianiManal MohammedAna CoelhoManel Ben HammoudaAna Cristina GonçalvesManfred P. LutzAna DascaluMangla SoodAna Djordjevic-DikicManikandan RamachandranAna Espinel-IngroffManindra TiwariAna I. ArrobaManish BhattAna Isabel Rocha FaustinoManish CharanAna Luiza De Carvalho FelippiniManish DhawanAna MarcãoManish RanjanAna Maria CastrucciManish Vijaya KumarAna Teresa Corte-RealManjur KolharAna Vujaklija BrajkovićManohar BhandariAnamaria BechirManoj SharmaAnamaria BrozovicManpreet SinghAnamaria SavuMansi SharmaAnanda MondalManuel Casal-GuisandeAnanda Shankar DattaManuel Duran-PovedaAnandharajan RathinasabapathyManuel Eduardo Vargas GuzmánAnanth S. EleswarapuManuel FuentesAnastasia MitseaManuel Gonzalez De La RosaAnastasia PapaporfyriouManuel GranellAnastasia ProdromidouManuel MadrazoAnastasia RakowManuel RitterAnastasios KoulaouzidisManuela ArbuneAnastasios KyriazoglouManuela Di FrancoAnatoliy BatyukManuela MatesanAnca BojanManuela MontanaroAnca ColitaMar Llamas-VelascoAnca MargineanMara CarsoteAnca PanaitescuMarc DeanAnca-Olguța OrzanMarc HilhorstAndaleb KholmukhamedovMarc PoulyAnders BergqvistMarc SarossyAnders Christian LarsenMarc-André LécuyerAndi RrokuMarc-Antoine De La VegaAndrada SeiceanMarcel BernucciAndras BanvolgyiMarcel SchmidtAndras BikovMarcella ZollinoAndraz DovnikMarcello BeneventoAndre De CarvalhoMarcello CandelliAndré TravessaMarcello CovinoAndre WirriesMarcello DemiAndrea AbateMarcello Di MartinoAndrea AlexandreMarcello RomanoAndrea AngeliniMarcelo ArancibiaAndrea BarisonMarcelo PalinkasAndrea BeccioliniMarcelo WerneckAndrea BoccatondaMarcia HiriartAndrea BorghesiMarcin A. WnukAndrea BuscaroliMarcin ArciszewskiAndrea CaraiMarcin DelijewskiAndrea CiminiMarcin DziekiewiczAndrea De VitoMarcin MaciejczykAndrea Dell’AmoreMarcin OpławskiAndrea GianniniMarcin SadowskiAndrea Gonzalez-MontoroMarcin WełnickiAndrea LisottiMarco Alfonso PerroneAndrea LoddoMarco BattistaAndrea Luigi Camillo CarobbioMarco CarmignaniAndrea M. QuattriniMarco ChiostriAndrea MoscatelliMarco CicconeAndrea MulliriMarco Di PaoloAndrea PiccinMarco Di SerafinoAndrea SalzanoMarco E. M. PelusoAndrea SambriMarco FilippucciAndrea SartoriMarco FoganteAndrea SonaglioniMarco GesiAndrea TinelliMarco La VerdeAndreas GantenbeinMarco ManciniAndreas LipphausMarco ManziAndreas LoeningMarco MorselliAndreas ManiosMarco MoschettaAndreas PeyrlMarco PortelliAndreas PolydorouMarco RoccettiAndreas PrachaliasMarco Stefano DemarchiAndreas PüspökMarco ZuinAndreas SchichoMarcos Arturo Martínez-BanaclochaAndreas SeitzMarcos BrioschiAndreas UntergasserMarcos Edgar HerkenhoffAndreas WarnkeMarcos Mello MoreiraAndrei Martinez-FinkelshteinMarcus BrooksAndrej ThurzoMarcus SchiltenwolfAndrés Alonso Agudelo-SuárezMare KoitAndrés Orozco-DuqueMarek FolAndrew C. BirkelandMarek KieliszekAndrew ChurgMarek MuriasAndrew ClarkMari SakamotoAndrew GrossbachMaria AlonsoAndrew J. BonhamMaria AmbrosioAndrew K. GormleyMaria Angela LosiAndrew KatsifisMaria Angeles Caballero-MoraAndrey ChernovMaria BarbolinaAndrey GrigorievMaria CappuccilliAndrey SimbirtsevMaría Carmen Blanco-LópezAndrey SudarikovMaria Costanza ChitiAndrey ZhelankinMaria Cristina Conti BellocchiAndrija MatetićMaria De Fátima Pereira Da SilvaAndrijana VčevaMaria E. GrigoriouAndrzej GrzybowskiMaria Elena MarcocciAndrzej KoniecznyMaria Elena RiccioniAndrzej KurylakMaria Elisa MancusoAndrzej KurylcioMaría Eugenia PortilloAndrzej MatiolańskiMaria FortofoiuAndrzej PolańczykMaria Francesca BaiettiAndrzej SlominskiMaria Francesca SfondriniAndy Wei-Ge ChenMaria GoulielmakiAneta AleksovaMaria Grazia MancinoAneta Cymbaluk-PłoskaMaria Isabella DoneganiAneta Ostróżka-CieślikMaría Jesús Alvarez CuberoÁngel Luis García-OtínMaría Jesús RodrigoAngel Robles-MarhuendaMaria João MenesesÁngela Ayén-RodriguezMaría José García-PolaAngela Dalia RicciMaria KallergiAngela MusellaMaría Laura GutiérrezAngela PapaMaría Lourdes Alarcón FortepianiAngela RizziMaría Orosia Lucha-LópezAngela SpanuMaria PalmieriAngelica GiulianiMaria Paola MogaveroAngelika GeroldingerMaria PaparoupaAngeliki AngelidiMaria PassanteAngelo BarbatoMaria Rita BiancoAngelo CastelloMaria RizziAngelo NaselliMaria Serafina RistaldiAngelo RegaMaria Serena ChiriacòAngelo RuggieroMarian SimkaAngelo TorrenteMariana BarbagianniAnibal CoronelMariana FloriaAniello MaieseMariana TudoranAnil Kumar JonnalagaddaMarianna MartinelliAnil TharappelMarianne BrodmannAnindit MukherjeeMariano CingolaniAnish BahraMariano SerraoAnita FekonjaMaribel Forero-CastroAnita Lyssek-BorońMarie BrandtAnita TurnerMarie Øbro FosbølAnja Kathrin JaehneMarie SüßeAnkit KumarMarieke StammesAnna BaraniakMariella DonoAnna BaritussioMarie-Luise KromreyAnna Bogusława Pilewska-KozakMarija IvanovAnna Boroń-KaczmarskaMarija ŽarkovAnna BoruckaMariko KawashimaAnna CapozziMarina LeiteAnna CieślińskaMarina MacchiaioloAnna DanielewiczMarina MartelloAnna ErmundMarina MuzzaAnna Giulia PavonMarina Šprem GoldštajnAnna Grzywa-CelińskaMarinela BostanAnna Jamroz-Wis̈niewskaMario AbateAnna Kablak-ZiembickaMario CrucianiAnna Kasielska-TrojanMario FranchiniAnna Lena FisseMario LimaAnna LewinskaMario MascalchiAnna Maria HeikkinenMario Mendoza-SagaonAnna Maria TestiMario MontaninoAnna MatyniaMário Pereira VéstiasAnna MeyfourMarion EveillardAnna N. BerlinaMarios G. KrokidisAnna Olasińska-WiśniewskaMarios KarvouniarisAnna OrłowskaMarisa GranatoAnna Paradowska-StolarzMarisa ShiinaAnna PecorelliMarish OerlemansAnna PisanoMarisol OcampoAnna PodlasekMarius BirleaAnna ProcopioMarius CrainaAnna Rita MigliaccioMàrius V. FuentesAnna SobiepanekMariusz KlenckiAnna Sołtysik-PiorunkiewiczMariusz SikoraAnna StarshinovaMariusz StasiolekAnna StarzyńskaMariya LivzanAnna V. MaslennikovaMarjolaine WillemsAnna Waśkiel-BurnatMarjorie JonesAnnalisa D’ArcoMárk AntalAnnalisa PaceMark BuzzaAnna-Maria BarciszewskaMark C. PreulAnnamaria NicolliMark J. RoefAnnarita NappiMark K. FarrugiaAnnarosa Del MistroMark S. ScherAnne SchützenbergerMark Ter LaanAnne-Laure RouxMarko BaškovićAnnelize McKayMarko KumrićAnnette B. PfahlbergMarko LucijanićAnnibale VersariMarko PanicAnnika HeuerMarkus LerchbaumerAnoop ArunagiriMarlena Zielinska-GorskaAnouar Ben MabroukMarsa GholamzadehAnpei YeMarshleen YadavAnthony Michael HunterMarta CalatroniAnthony T. RederMarta Diaz-DelcastilloAnthony William ColemanMarta Elena Losa IglesiasAnthoula GaigneauxMarta F EstradaAn-Ti ChiangMarta FijałkowskaAnton KrivopalovMarta FocardiAnton Pavlovych GerilovychMarta LlopAnton StaudenherzMarta MazurAntonella ArgentieroMarta Prieto VicenteAntonella CecchettoMarta RusekAntonella CurulliMarta SkowrońskaAntonella FioravantiMarta Waliszewska-ProsółAntonella MeloniMartha Patricia Gallegos-ArreolaAntonella TomassettiMartín Alberto BelzunceAntonia Dimitrakopoulou-StraussMartin ChanAntonija TadinMartin E. BlohmAntonina ArgoMartin GottliebsenAntonino Lo GiudiceMartin HartrumpfAntonio BarileMartin HelanAntonio BellastellaMartin JaegerAntonio BiondiMartin KeuchelAntonio BulumMartin LoertscherAntonio C. P. WongMartin MischAntonio CaruzMartin RössleAntonio CicioneMartin SiglAntonio Córdoba FernándezMartin VilligerAntonio CorteseMartin ZachariasAntonio FacciorussoMartina MaggiAntonio GidaroMárton SzemenyeiAntonio GiordanoMartti VastamäkiAntonio GomezMaruška MarovtAntonio GranataMaruti Ram GudavalliAntonio MacciòMarwa GaberAntonio ManentiMary Garland-KledzikAntonio MassaroMary M. SalvatoreAntonio Mendes-SousaMary N. SheppardAntónio Miguel MorgadoMaryam ArdalanAntonio R. Hidalgo-MuñozMaryam RavanAntonio Ramos MartinezMaryem RhanouiAntonio RizzaMarzia SegùAntonio SanzoMarzieh SalimiAntonio ScaranoMarziliano NicolaAntonio Simone LaganàMasaaki KusunoseAntonio VallanoMasaaki ShimataniAntonio VilasiMasafumi OdaAntonios SiderisMasahide OkiAntonios TzortzakakisMasahiko SugimotoAnum RahmanMasahiro SaitoAnúp PandíțhMasahito FushimiAnuradha BhukelMasakatsu TsurusakiAnusak KerdsinMasakazu YamadaApostolos KarantanasMasaki OkamotoApostolos ZaravinosMasaki YasufumiApurva Tadimari PrabhakarMasanao NakamuraAram KimMasanobu IbarakiAram YangMasao KobayakawaArash HeidariMasaomi YamaneAravinthkumar JayabalanMasaru TanakaArchana MishraMasashi NinomiyaArchana PrabaharMasataka KikuyamaArchi Ramesh AgrawalMasato YonedaArgiris S. SymeonidisMasatoshi HottaArgyrios PetrasMasayuki FujiwaraAria BaniahmadMasayuki TanabeAriadni SpyroglouMasayuki TsunekiArieh IngberMasiero StefanoAriel FurerMassimiliano AragonaArif SiddiquiMassimiliano De PaolisArifin Budiman NugrahaMassimiliano Di PietroAristeidis H. ZibisMassimiliano EspositoAristidis GaliatsatosMassimiliano OrsiniArkadiusz GertychMassimiliano TognoliniArkadiusz MatwijczukMassimo CapocciaArkady VoloshinMassimo ChelloArlindo SilvaMassimo CorsaliniArmando CavalloMassimo IacovielloArmando PatrizioMassimo LibraArminda Manuela GonçalvesMassimo SalvatoriArnaldo BatistaMassimo SalviArnaldo ScardapaneMassimo TusconiArnaldo StanzioneMatas JakubauskasArnaud AlvesMatei BratuArnoldo RiquelmeMateusz KoteckiArthur AndréMateusz SpiewakArthur B. CummingsMateusz WichtowskiArthur P. WunderlichMateusz ZawadkaArtit JirapatnakulMathew BloomfieldArtur Jorge FerreiraMathias C. BrandtArtur KrzyżakMathias TrempArtur PałaszMathieu GendrotArtur SłomkaMathilde GavilletArtur Tadeusz KrzyżakMatías Gastón PérezArturo BonomettiMatias IglickiArun RajasekaranMats ErikssonArūnas LukoševičiusMats W. JohanssonArun-Kumar Kaliya-PerumalMatteo BaucknehtArutselvan NatarajanMatteo Bruno LodiArvind Kumar ShuklaMatteo Cotta RamusinoAshis MondalMatteo FontanaAshish ChandraMatteo FusagliaAshish Kumar BhandariMatteo GhilliAshkan ZandiMatteo MontiAshok PanchapakesanMatteo NeriAshu JohriMatteo RenzulliAshutosh Dhar DwivediMatteo VittoriAshutosh SharmaMatthew A. TylerAsif KhanMatthew DonaduAsker KhapchaevMatthew FreemanAsma KhoobiMatthew L. FaronAstrid K. LeckMatthew M. WillmeringAsuman GedikbasiMatthew PainschabAtchara PhumeeMatthew Q. MillerAtefeh AbediniMatthew R. BurasAthanasia PapazafiropoulouMatthew SmithAthanasia PrintzaMatthias EberhardAthanasios AnagnostisMatthias FrölichAthanasios KiourtisMatthias NiedrigAthikhun SuwannakhanMatthias PriemelAthina MarkouMatthieu BaillyAtiqa AmbreenMatthieu GrohAtsuhiko YagishitaMatthieu PeycelonAtsuki NaraMatti HuotariAtsushi IkedaMatti Iso-MustajärviAtsushi K. KonoMattia BellanAtsushi SakamotoMattia MiottoAtsushi TeramotoMattia RiefoloAttila FrigyMattis GeigerAttila Gergely VéghMaurice MarsAttila KovácsMaurizio BalbiAttila OlahMaurizio DomaninAudi Francesca SetiadiMaurizio MargaglioneAugust WrotekMaurizio MartiniAugusto FuscoMaurizio PolanoAurel Popa-WagnerMauro AlaibacAurelia Cristina NechiforMauro GiacomelliAurelio Luna MaldonadoMauro GiuffrèAurora ArghirMauro MastropasquaAustin L. LaGrowMauro PepiAwadhesh KumarMax HeilandAwanish MishraMax MenziesAwatef KelatiMaxime PichonAxel BoeseMaximilian SchmidtAxel KünstnerMaximilian ZeydaAxel SchmutzMay BisharatAyako MatsudaMazen SalehAydin EresenMazhar Javed AwanAyman AlthuwaybMazin Abed MohammedAyman El-HattabMd Alamgir KabirAyman ElnahryMd Junayed HasanAysegul YildizMd Mamunur RahamanAyşegül YurtMd Manjurul AhsanAytaç AltanMd Robiul IslamAyush DograMd. Alamgir HossainAzamat TemerdashevMd. Shahid SarwarAzhar IlyasMd-Zahid AkhterAziah DaudMegha SinghalB. Naresh Kumar ReddyMehboob HoqueBabett RamsauerMehdi HassanBagus Setya RintyarnaMehedi MasudBahattin Kerem AydınMehmet Ali ErgünBahil GhanimMehmet GülBaijian YangMehmet KocaogluBaki HekimoğluMehmet SatarBalachandra Suryakant AnkadMehrez ElnaggarBalakrishna KoneruMeinrad BeerBalik DzhambazovMeixue DongBanghyun LeeMelania MaglioBaohui XuMelanie C. Alonzo MartínezBarbara BertoglioMelinda Y. ChangBarbara BrognaMelisew Tadele AlulaBarbara FazekasMelissa Agsalda-GarciaBarbara J. FuegerMelissa L. HillBarbara JasiewiczMelissa Orzechowski XavierBarbara MognettiMenelaos ZafrakasBarbara MulloyMeng LiuBarbara PalumboMeng-Che HsiehBarbara Ruszkowska-CiastekMengchu ZhouBart De GeestMengxi ShenBart PijlsMengyu WangBart VandenbergheMercedes Mitjavila-CasanovasBartłomiej Henryk NoszczykMercedes MuñozBartosz MałkiewiczMeredith E. PittmanBashayer Al-MubarakMériam SabbahBasilio VescioMervyn J. EadieBastian SabelMete YağanoğluBayodé Roméo AdégbitèMichael BartelsBeáta Grešs HalászMichael BermanBeata Kasztelan-SzczerbinskaMichael CottonBeata Labuz-RoszakMichael DevaneBeata Sarecka-HujarMichael FleischhackerBeatrice KnudsenMichael G GravettBéatrice W. KirubiMichael Glen BeckerBeatrix Cochand-PriolletMichael HeneinBeda HartmannMichael J. SinghBegoña Acha PiñeroMichael KnitschkeBegoña González-SuárezMichael KochBehrooz H. YousefiMichael MorganBehzad FarahaniMichael Nair-CollinsBen Z KatzMichael O’ConnorBenedetta GuidiMichael PretterklieberBenedetto FalsiniMichael SchirmerBeniamin Oskar GrabarekMichael SticherlingBenjamin BlümelMichael WeismanBenjamin CullMichael WelshBenjamin L. MaughanMichael William MatherBenjie TangMichaela CellinaBernadette CoricaMichail E. KlontzasBernard SpeculandMichał GontarzBernard Ten HakenMichal Herman-EdelsteinBernardo Sousa-PintoMichal KouckyBernardo VegaMichal KovoBernd FinkMichał Krzysztof KowaraBernhard RauchMichal LechBernhard SchwabergerMichal MinarBernhard ZelgerMichał PomorskiBert De VriesMichal SarulBert VandenberkMichał StrzeleckiBerthold HocherMichał ŻorniakBertrand TchanaMichalina BłażkiewiczBettina BlaumeiserMichel NieuwoudtBettina CarvalhoMichel RigaudBetty Yuen-Kwan LawMichel RoethlisbergerBhanu TewariMichela FerrucciBharat GuptaMichela FranchiniBharath Babu NunnaMichela GabelloniBharathi DevarajMichela GrossoBhawna DevMichela QuadriniBhawna NigamMichelangelo LucianiBhisham SharmaMichele CorrealeBhola PradhanMichele Cosimo SantoroBiagio BaroneMichele Del ZingaroBiagio DidonaMichele Di CosolaBiagio RaponeMichele FioriniBianka ForgoMichele GolinoBianka HeilingMichele KlainBin QiuMichele OrtolaniBin SongMichele PietragallaBing GuMichelle A. EspyBinsen LiMichelle MellenthinBirgit Arnoldt-SchmittMieszko WięckiewiczBiswajit PadhyMiguel A. OrtegaBjörn KemperMiguel AbalBo WangMiguel AlcarazBoban M. ErovicMiguel Ángel González GallegoBogdan BatkoMiguel Ángel Torralba-CabezaBogdan DorofteiMiguel Bravo-ZanogueraBogdan F. IliescuMiguel GoncalvesBogdan GhermanMiguel Gonzalez-MuñozBogdan Silviu UngureanuMiguel HuesoBogdan SoceaMiguel IdoateBogdan TiguMiguel Malo-UrriésBojan V. StimecMihaela HostiucBojan ZaricMihaela IlieBojana N StamenkovicMihaela IonescuBooma Sowkarthiga BalasubramaniMihaela TurtoiBorek SehnalMihaescu GrigoreBo-Young HongMihai CapilnaBoyoung JoungMihai Dorin VartolomeiBożena SobkowiczMihai JuncarBrad BarnettMihai PopescuBradford K. BergesMihai RomanBram JacobsMihai ZahariaBrandon Lucke-WoldMihi YangBranka Petrić-MišeMiihir Narayan MohantyBranko KolaricMikhail G. AkimovBrant G. WangMikhail TarkovBratko FilipičMiki KushimaBrega CarlottaMikkel NoerholmBrent SieskyMilad ZandiBrian BalogMilan JakubekBrian BrownMilan LepicBrian CrookMilan PrsaBrian G. BoothMilan SkitekBrigitta G. BaumertMilan TomaBrigitte BruijnsMilena Magalhães AleluiaBrittany MurphyMilica DeklevaBritt-Isabelle BergMilica PopovicBruna VisniauskasMilica ZekovicBruno BalbiMiłosz CabanBruno ChrcanovicMilosz JankowskiBruno FiondaMin ChenBruno MendesMin Gyu KyungBruno PassarettiMin Kyu KangBruno TrimarcoMin ParkBryan M. WongMin PengByoung Kuk JangMin XieByoung-Hee LeeMina MahdianC. Burton WoodMinato YokoyamaCaitlin SchneiderMinerva Codruta BadescuCălin CăinapMing ChenCalin CorciovaMing Shiuan SohCalin VaidaMing ZhaoCalogero CasàMing-Chih LinCalogero DiBellaMingchung ChouCalogero VetroMinghang WangCamelia CojocariuMinghao GongCameron MillarMinglei ShuCamilla KärnfeltMing-Li HsiehCamilla S. GrahamMingsheng ChenCamille DesgrouasMing-Ting WuCandice BremMinhao XieCarla Maria Batista Ferreira PiresMinjuan ZhengCarlo AlfieriMinli YouCarlo AprileMinoru MiyazatoCarlo Augusto MallioMinoru SatohCarlo BertoncelliMio EbatoCarlo BizMioara BercuCarlo CaiatiMiran ČokloCarlo CavaliereMircea Ioan PopaCarlo De AsmundisMircea-Catalin FortofoiuCarlo PallottoMireia Bolívar-PradosCarlo PerisanoMirela Cleopatra TomescuCarlo RodellaMirela GhergheCarlo SaccardiMirella MarinoCarlo TrombettaMiriam BoppCarlos Alexandre Gouvea Da SilvaMiriam MenzelCarlos Bernal-UtreraMirjam GerwingCarlos Eduardo BaccinMirko ManettiCarlos Eric Galván-TejadaMirko Parasiliti-CaprinoCarlos Francisco Simões GomesMiro JukićCarlos G. JuanMiroslav RadenkovicCarlos Garaicoa-PazminoMiroslava NedyalkovaCarlos GasparMirosław CzuczwarCarlos Hernando GómezMirosław ŚwierczCarlos Ignacio Ponte-NegrettiMiroslaw WielgosCarlos LoncomanMirza PojskićCarlos López-de-CelisMisao NishikawaCarlos Martin LlorenteMislav MikusCarlos Roberto GrandiniMislav RadicCarlos Roberto Hall BarbosaMithalesh Kumar SinghCarlos Rocha-De-LossadaMitra AkbariCarlos Torres-TorresMitsuaki IshidaCarlton DampierMitsuhiko OsakiCarmela NappiMitsuo MotobayashiCarmela PaolilloMitsuru NakazawaCarmela RinaldiMitsuru SugimotoCarmelo CorsaroMitsushige SugimotoCarmen CadillaMizuho NishioCarmen Cámara HijónMladen AndjićCarmen De MendozaMladenko VasiljevićCarmen Dora Méndez-HernándezMobeen RehmanCarmen Guillén-PonceMoez KrichenCarmen Méndez HernándezMohamed Abd ElazizCarmen Moret-TatayMohamed Adel HammadCarmen Patino-AlonsoMohamed Al-ShabraweyCarmen PredaMohamed BahajCarmen SavinMohamed MahdiCarmen SerranoMohamed MeghilCarmen SolcanMohamed SolimanCarmine FinelliMohamed Yaseen JabarullaCarmine IzzoMohammad AbdolahadCarmine PiconeMohammad Abu YousufCarmine PizziMohammad Akhlaquer RahmanCarolina Beatriz TabernigMohammad AlamCarolina EscuderoMohammad Al-HatamlehCarolina S. VinagreiroMohammad Arif Sobhan BhuiyanCarolina SolomonMohammad Bagher KhodabakhshiCarolina VerissimoMohammad El KhatibCaroline HastingsMohammad Enamul Hoque Kayesh KayeshCarsten BalczunMohammad HajeerCarsten CarlbergMohammad HashempurCatalin StoeanMohammad Hassan HodrojCatalin TiliscanMohammad JamshidiCatalina AvendañoMohammad KhisheCatalina MihaiMohammad Mahbubur Rahman Khan MamunCatarina FerreiraMohammad Mehdi RoshaniCatarina GouveiaMohammad Mehedi HasanCatarina RuaMohammad MoghimiCaterina FedeMohammad MohammadianpanahCaterina LeddaMohammad MohsenzadehCatherine Weikart YeckelMohammad ShahbakhtiCatherine-Isabelle GrosMohammad ZamaniCécile ManceauMohammad Ziaur RahmanCecilia Alegado JimenoMohammadreza ShalbafanCecilia Di RubertoMohammed DiykhCecilia VarjuMohammed HadwanCecy Martins SilvaMohammed MecheeCédric LenormandMohammed NoushadCelia Sánchez-RamosMohammed RohaimCemil ColakMohammed SallahCengiz OzalpMohammed Yacin SikkandarCésar Calvo-LoboMohan Kumar KalaiahCesar TorresMohd Suhail AkhterCh GangadharMohd Zulfaezal Che AzeminChae Moon HongMohit JoshiChahnez Charfi TrikiMojgan AlaeddiniChaido SirinianMominur RahmanChakfong CheangMomoko TsudaChander Shekhar MukhopadhayayMona Abdelbaset Sadek AliChandrakala Aluganti NarasimhuluMona DabiriChandrakanta MahantyMona VintilaChandramani PathakMonali SwainChang Ik YoonMonica BalzarottiChang-Hoon ChoiMonica FiraChang-Shen LinMonica HernandezChangshin KangMónica MartinsChangwon JeongMonica VisintinChao SongMonika KijewskaChaoyang GongMonika KoziełChao-Yu GuoMonika RacChara GeorgatzakouMonika SkorupaCharalampos N. PitasMonika SzturmowiczCharalampos SiristatidisMonique Courtade-SaïdiCharat ThongprayoonMoon Young KimCharles Joel RosserMoraitou DespinaCharles TruilletMoreno LelliCharles William YoxallMoreno ZanardoChee Meng Benjamin HoMorgan BroggiChellappagounder ThangavelMorihito TakitaChen ZhuMoses OkpekuCheng MaMoses SamjeCheng XueMossab Y. SaeedChengcheng LiuMouadh BarbirouCheng-Hsun HoMrigya BabutaChenglei LiMuahammad IslamCheng-Maw HoMubarak MashrahCheng-Yu LongMubarak Taiwo MustaphaChen-Xiong HsuMugurel Constantin RusuChe-Yu LinMuhammad AlsherbinyChi ZhaoMuhammad ArsalanChia-Jung ChangMuhammad AtifChian Ju JongMuhammad Atif ZahoorChiara Da PieveMuhammad Attique KhanChiara Di TucciMuhammad Fayyaz ur RehmanChiara LiveraniMuhammad Fazal IjazChiara Maria MazzantiMuhammad ImranChiara MartiniMuhammad Imran RahimChiara ScapoliMuhammad KamranChiara VeneroniMuhammad KhurramChia-Yuan ChangMuhammad NazirChi-Ching ChangMuhammad SardarazChien-Chang ChenMuhammad SyafrudinChien-Chang HsuMuhammad UmairChien-Feng LiMuhammad Umair AliChien-Hung ChenMuhammad Usama IslamChien-Hung LiaoMuhammad Usman MunirChien-Ming ChaoMuhammad Usman TariqChien-Tai HongMuhammad ZeeshanChien-Te LeeMuhammad ZubairChih-Cheng LaiMuhammed UnlersenChih-Ching LinMuhammet BaykaraChih-Jen YangMuhammet Fatih AslanChih-Li LinMuhibUr RahmanChih-min TsaiMukesh KumarChihwen WangMukherjee SaswataChih-Yang WangMukul VijChi-Ju KimMuneeswar NittalaChi-Ming WongMurad HossainChin-An YangMurat AladaǧChing-Hua HsiaoMurthy ChavaliChing-Sheng HsuMushriq AbidChing-Ta LuMustafa Berkan BicerChing-Wei WangMustafa CesurChingyu LinMustafa SevindikChinmaya MahapatraMustafa SirlakChin-Mu HsuMuthu SathishChinthaka B. SamaranayakeNaazneen MoollaChin-Yu LinNadia NathanChiranji Lal ChowdharyNadine BorstChiranjibi SitaulaNadine S. AguileraChiuan-Chian ChiouNadira DurakovićChiung-Mei ChenNadira KarunaweeraChong ChenNadja EngelChongwen WangNagur Shareef ShaikChow Wei TooNagwan SalehChoy Ker WoonNaji KharoufChris Fife-SchawNajib HaboubiChris Hong Long LimNalu T. A. PeresChristelle YeromonahosNami OhashiChristian A. MuellerNan YangChristian BarbatoNan ZhaoChristian De GeyterNana Kwadwo BiritwumChristian HeegerNancy E. KemenyChristian J. KellenbergerNandaraj TayeChristian Manfred HammerNaoharu YagiChristian Mata MiquelNaoki HosoeChristian RoquesNaoki MatsumotoChristian Scheurig-MuenklerNaoki SakataChristian SturessonNaoki UmemuraChristian TescheNaoki YamamotoChristian WirajaNaoko OkanoChristiana KyvelidouNaomi A. PorretChristina Gerth-KahlertNaoro TanakaChristina MejersjöNaotaka NittaChristine HoeffelNaozumi IshimaruChristodoulos E. PapadopoulosNarendiran RajasekaranChristof MittermairNaresh Kumar RavichandranChristof RottenburgerNaseer AhmedChristoforos KosmidisNaside MangirChristoph F. DietrichNatalia BelosludtsevaChristoph FaschingerNatalia GrubaChristoph GrossNatalia I. KuryshevaChristoph PalmNatalia IssaevaChristoph PeterNatalia KreshchenkoChristoph ThalhammerNatalia MadetkoChristoph TrummNatalia RiveraChristophe MarzacNatalia TibertiChristophe PrehaudNatallia V. DubashynskayaChristopher BallmannNatalya GlushkovaChristopher BoyleNataša KnapChristopher E. BauerNataša RančićChristopher EalandNataša S. StanisavljevićChristopher J. L. NewthNathalie VermeulenChristopher J. YoungNathan K. EvansonChristopher KlothNathan MewtonChristopher KobierzyckiNatheer KhasawnehChristopher M. KappNattapol AunsriChristopher PayanNaveed Ullah KhanChristopher R. SnellNavid AbedpoorChristopher WardellNavid RabieeChristos ArnaoutoglouNavid RazmjooyChristos K. KontosNazefah Abdul HamidChristos K. YiannakopoulosNazia HameedChristy L. PylypjukNeda Haj-HosseiniChrysanthi KarapantzouNeda SladeChrysovalantis KorfitisNeema MoravejiChuan Hun DingNeeraj SharmaChueh-Hung WuNeftali Eduardo Antonio-VillaChun Hung ChuNeha KoonjooChunchia ChengNehal ElsherbinyChun-Fu LiuNeil GlossopChung-Chien HuangNélio VeigaChung-Hui LinNemanja JovičićChung-Jung LiuNemanja MačekChung-Lieh HungNenad BukvicChung-Lin LeeNenad FilipovicChung-Min WuNesrein HashemChung-Ta LeeNguyen Minh DucChun-Gu ChengNguyen Quoc Khanh LeChunqi QianNicholas Faure WalkerChun-Te WuNicholas FischerChunyi WuNicholas P. RowellChun-Yu LinNico BulsCid André Fidelis De Paula GomesNico MelzerCinzia MasperoNicola CavasinCiprian RezusNicola F. FletcherCiprian TomuleasaNicola K. DinsdaleCiro Gargiulo IsaccoNicola Luigi BragazziCiro ImbimboNicola MangialardiCiro SantoroNicola MarottaClaire L. JacksonNicola MontemurroClara HjalmarssonNicola MumoliClaude Van CampenhoutNicola ZampieriClaudia Angela Di SilvestriNicolae BacalbaşaClaudia DittfeldNicolae BacalbașaClaudia LoretiNicolae GicaClaudia MehedintuNicolae Popescu-BodorinClaudio CelentanoNicolas BruderClaudio GambardellaNicolas ChapelleCláudio MaiaNicolas GauvritClaudio ZuccaNicolas GolseClaudiu MargineanNicolas LejeuneClaus Bo SvendsenNicole RübsamenClément MarcelinNicoleta AntonClément PicardNicolò BizzarriClément R. PicardNicolò CardobiCleverton De AndradeNicolo De PretisCliff Richard KikawaNicolòmaria BuffiClifton AddisonNicos MaglaverasClinton WebbNidhi SinglaCodruta SoicaNiels QvistCody AshbyNigel RobbConnah KendrickNighat NoureenConstantin T. LucaNihan AydemirConstantina AggeliNiki KatsikiConstantino Carlos Reyes-AldasoroNiklas PakkasjarviConstantinos DemonacosNikola AnđelićCorine Geurts KesselNikola BatinaCorrado CiattiNikola PopovićCorrado TagliatiNikolai Andreevitch SobolevCorsino Rey GalánNikolaos A. ChrysanthakopoulosCosimo Di RaimondoNikolaos AndreatosCosimo SpertiNikolaos MachairasCosmas Ifeanyi NwakanmaNikolaos PistamaltzianCosmin CituNikolaos StalikasCosmin EneNikolaus B. BinderCosmin PuiaNikolay KazanskiyCostantina ProtaNikolay RubtsovCostantino CiallellaNikoleta PrintzaCostantino MancusiNikos PetrellisCostas S. ConstantinouNikos ViazisCristian CastilloNilanjan DeyCristian FurauNilson Ivo Tonin ZanchinCristian Iulius SurcelNina GolyandinaCristian Prieto-GarciaNiranjana SampathilaCristian RotariuNirmal Kumar MohakudCristian ScheauNirmal MazumderCristian StătescuNisarat PhithakwatcharaCristiana Iulia DumitrescuNitin AmdareCristiana VoicuNitin TelangCristiane GuzzoNitish KatalCristiano CarbonelliNivedita GuptaCristina GiannattasioNizar TliliCristina GluhovschiNoboru OriuchiCristina Manuela DrăgoiNobuhiro YodaCristina MargineanNobumichi KobayashiCristina Martin-SabrosoNobuto NakanishiCristina NadaluttiNoé Macias-SeguraCristina TudoranNoemi DirzuCsaba OláhNoemí López-EjedaCsilla CzimbalmosNoha Mousaad ElemamCurtise Kin Cheung NGNoor Zaman JhanjhiCynthia A. ProwsNorbert KissCynthia L. BodkinNorbert WeilerCyrille BlondetNorihiko NaritaCyrus MotamedNorihiro NishidaDafna SussmanNorio MiyoshiDagmar PavluNorio YamamotoDai D. NghiemNorman Scott LitofskyDaiji NagayamaNorman Van RhijnDaimantas MilonasNoura DosokyDaiqin ChenNoureddin Nakhostin AnsariDaisuke AkagiNunzio CirulliDalia MedhatNurdan AcarDalin ZhangNuryani NuryaniDamien CostaNuttada PanpradistDamir FabijanićNuttha LumlertgulDamjan GlavačNydia Tejeda MunozDan ChenOana SandulescuDan Cristian RaduOctavian EnciuDan QiOgnjen BarcotDan VoiculescuOhsawa SuguruDana Carmen ZahaOhsuke MigitaDana CramariucOke GerkeDana StoianOkyaz EminagaDanail PavlovOlaf GefellerDanala GopichandhOleg O. MyakininDane KrtinicOleh BerezskyDanfeng SunOleksandr GrynkoDang Ngoc Hoang ThanhOleksii SkorokhodD'Angelo C. MaglianoOleksii TyshchenkoDania CioniOlexander BarmakDaniel BiermannOlga AnatskayaDaniel BoegerOlga CiepielaDaniel ChristophOlga GutkowskaDaniel DawsonOlga KruszelnickaDaniel LighezanOlga Mikhailovna BazanovaDaniel Loïc PouliquenOlga NetskinaDaniel López MaloOlga RedinaDaniel López-LópezOlga Y. KorolkovaDaniel Marcelo CisternaOliver HanemannDaniel NeureiterOliver SanderDaniel P. PotaczekOlivier ProuxDaniel Pérez-PrietoOlivier RukundoDaniel PinkOluwatosin OluwadareDaniel R. GangerOm Prakash ChoudharyDaniel Robert SchlattererOmar Yassef Antùnez-MontesDaniel RomeroOmbretta TurrizianiDaniel SalamangoOmid HamidiDániel SandiOmneya AttallahDaniel ŚlęzakOmnia HamdyDaniel TurudićÖnder ErgönülDaniel Vasile BalabanOndřej PetrákDaniel W. NixonOnur ErgenDaniel Wai-Hung HoOPhir NaveDaniela CarmagnolaOriana TrubianiDaniela Di VenereOrietta PicconiDaniela Maria TanaseOrlando Aguirre GuedesDaniela MarzioniOrlando Guntinas-LichiusDaniela Opris-BelinskiOrnella CominettiDaniela TerraccianoOsagiede OsayandeDaniele CorboOsamu YamaguchiDaniele Della LattaOsbourne QuayeDaniele DiacintiOscar F. AranedaDaniele GarcovichOscar J. PerdomoDaniele GiansantiOscar Martínez-PérezDaniele Giovanni GhiglioniOskar SachenkovDaniele MasaroneOskars KalejsDaniele NovielloOsmindo R. Pires JúniorDaniele OrsoOttavia D'OriaDaniele PironiOvidiu PopDaniele SolaOxana DobrovinskayaDaniele TognettoOzgur Koray SahingozDaniele Ugo TariOzren PolasekDaniele UrsoOzren PolašekDaniella PrayerÖzüm Yuksel TunçyürekDanielle K. DeperaltaPablo A. KlerDaniel-Mihai RusuPak Hin Hinson CheungDaniil N. BratashovPakkasjärvi NiklasDaniil P. AksenovPallavi GuptaDanijela Đukić-ĆosićPamela PinzaniDanijela RadojkovicPamela TozzoDanijela Vrdoljak-MozetičPan ZhangDanilo BuonsensoPanagiotis AthanassiouDanilo LofaroPanagiotis F. LiaparinosDanuta Gąsior-PerczakPanagiotis G. HalvatsiotisDanuta Lietz-KijakPanagiotis KaranisDaoying GengPanagiotis KorovessisDaphna Mezad-KourshPanagiotis MallisDardan KlimentaPanagiotis PeitsidisDaria AugustyniakPanagiotis Radoglou-GrammatikisDariga AkashevaPanagiotis TheodorakisDar-In TaiPanagoula KolliaDarin ZertiPanayiotis KouisDario CalafiorePanayiotis LoukopoulosDario FajPang-Yun ChouDariush IraniPanisadee AvirutnanDariusz NowakowskiPaniz JasbiDarwin CastilloPanos KosmasDavid BalgomaPaola FeracoDavid C. RotzingerPaola GargiuloDavid C. StiegPaola MapelliDavid DannhauserPaola ParenteDavid FuksPaola ParlantiDavid KachlikPaola QuaresimaDavid KipgenPaolo AngeliniDavid M. AllenPaolo AseniDavid MoleroPaolo BennaDavid PollyPaolo BrusiniDavid RequenaPaolo CampioniDavid RichardsPaolo CanepaDavid RockabrandPaolo CapparèDavid WelchPaolo CavorettoDavid WinkelPaolo DolzaniDavid ZemanPaolo FormentiDavid ZweikerPaolo FrassanitoDavide BorroniPaolo ImmovilliDavide CapponPaolo Maria LeoneDavide FirinuPaolo MarraDavor PlavecPaolo MartellettiDawid SzwedowskiPaolo MonardoDa-Yuan ChenPaolo RadiceDebananda GogoiPaolo SpinnatoDebasish Kumar DeyPaolo VersacciDeborah BrownPaolo ViscontiDebsindhu BhowmikPaolo ZaffinoDeepak KulkarniPaolo ZamboniDeepak Kumar KhajuriaPapangkorn InkeawDeftereos SavasParaskevas GkolfakisDeirdre DonnellyParaskevas LybérisDejan GeorgievParaskevi D. TheocharisDejan M. MicicParaskevi TrivilouDelio José MoraParastoo FarniaDemetra SocolovParisa GazeraniDemetrios ArvanitisParisa NaraeiDemetrios J. HalazonetisParita Rajiv OzaDenes TothParracino AntoniettaDengchao CaoParthasakha DasDeng-Guang YuParvathaneni Naga SrinivasuDenis SerenoPascal IckrathDennis AdjepongPascal LambertDennis FlanaganPaschalis TheotokisDennis Kai Ming IpPasquale CaldarolaDer-Shin KePasquale CianciDerya BirantPasquale FlorioDevis BenfaremoPasquale PisapiaDevojit Kumar SarmaPasquale VitoDhananjay YadavPasqualino SirignanoDharmendra ChoudharyPatricia N. ToninDi ZhangPatricia Switten NielsenDiana Angelika OlszewskaPatrick G. BradyDiana Luminita ManolescuPatrick HaDiana M. LindquistPatrick KwongDiana Marcela CastilloPatrick ShannonDiana MartinsPatrizia LoPrestiDick IanPatrizia ZaramellaDiego De OrtuetaPatrizio MazzoneDiego Dos Santos FerreiraPau Cerdà SerraDiego ViasusPaul E. SijensDieter MetzePaul Eric DossouDihogo De MatosPaul GardDilber OzsahinPaul GrossfeldDiletta CozziPaul L. CrispenDilip BagalPaul Le TurnierDima SabbahPaul SchoenhagenDimitra KoumakiPaul ValleDimitri PoddighePaula Juiz-ValiñaDimitrios KaragiannakisPaula TavaresDimitrios PatouliasPaulina AdamskaDimitrios VlachodimitropoulosPaulina DumnickaDimitris A. TsitsikasPaulina ŻarnowiecDimitris LambrouPauline HarperDimitris TatsisPaul-Mihai BoarescuDimitris TsakogiannisPaulo AlvesDimitris ZacharoulisPaulo CoelhoDina IbrahimPaulo Magno Martins DouradoDinesh KumarPaulo SantosDinesh Kumar VermaPaulraj KanmaniDinesh RokayaPavel DundrDiogo Telles-CorreiaPavel MatreninDionisio CarvalhoPavel OtrisalDionysios TafiadisPavol SzomolanyiDionysios VorosPaweł DolibogDirk RueterPaweł GaćDirk Van De PuttePawel KleczynskiDivya SinhaPaweł LinekDjamel DabliPawel MacekDmitri ShchekochikhinPawel MiotlaDmitrii S. MaltsevPaweł OlczykDmitrijs BliznuksPaweł RussekDmitriy Nikolaevich AndreevPaweł RynioDmitry A. GryadunovPayam BehzadiDmitry FrankPedro AmaroDmitry KhochenkovPedro GuimarãesDmitry SkvortsovPedro Manuel Moreno-MarcosDoerte WichmannPedro Vieira Da Silva MagalhaesDohern KymPei-Chen LiDoina A. TodeaPeide WuDoina AzoicaiPei-Fen YenDomen PlutPei-Yuan SuDomenica MangieriPeng GuoDomenico AlbanoPeng WeiDomenico CucinottaPengfei CaiDomenico Umberto De RosePengfei ZhangDominic OliverPenny A. McKelvieDominic Selorm Yao AmuzuPer Lav MadsenDominik DłuskiPer LinqvistDominik PruskiPer NordinDominik SaulPerez-Valle ArantzaDominika PohlmannPernille HolmagerDomiziano TarantinoPeršec JasminkaDomonkos VargaPetar ĐanićDonato D’AgostinoPetar OzretićDong Keon YonPeter BottenbergDon-Gey LiuPeter CelecDonghoon YoonPéter CsécseiDong-Kyu KimPeter DurdikDongxu ChenPeter GroenenDonovan McGrowderPeter HegyiDorin MoldovanPeter HermannDoris Yin Ping LeungPeter HollanderDorota Bielinska-WazPeter KorstenDorota Piotrowska-KownackaPeter KraiczyDorota Pojda-WilczekPeter MwangiDorte Bek FolkvardsenPeter NiedbalskiDouglas E. NeyPeter PaalDouglas R. WilsonPeter PensonDoychin AngelovPeter PikoDoyoung KimPeter SmithamDragana GabrićPeter VelthuisDragana M. StanojevicPeteris StradinsDragos Catalin JianuPether JildenstalDragos CozmaPetr DráberDragos SerbanPetr O. IlyinskiiDubravko HabekPetra KoraćDuilio DivisiPetra PetranovicDuminuco AndreaPetrick Ramesh K. PeriyasamyDumitrescu CatalinPetrik GalvosasDurmuş KoçPetros IoannouDusanka ObradovicPetros MouratidisDuska Martinovic KaliternaPetros PapalexisDustin FlanaganPhilip A. RubiniDzati Athiar Bt RamliPhilip Julian BroserE. Scott SillsPhilip V. GardinerEbenezer EsenoghoPhilipp AichingerEbrahim Mohammed SenanPhilipp FerversEbrahim NajafzadehPhilipp GensekeEbrahimi MehdiPhilipp WollmannEdgar Fabián Manrique-HernándezPhilippe A. GrangeEdith ClarosPhilippe PérotEdith LahnerPhilippe PetitEdmunds ReineksPhilippe SoyerEdoardo BraunerPhilippos KlonizakisEdoardo CarrettoPhitchayapak WintachaiEdoardo Gioele SpinelliPia Karlsland AkesonEdoardo MazzucchiPia Lopez-JornetEdoardo PasquiPiera Anna MartinoEduard GuaschPierfrancesco FrancoEduardo Arellano-RodrigoPiergiorgio DanelliEduardo José Veras LourençoPierluigi MarzuilloEduardo Pandolfi PassosPiero PavoneEduardo PimentaPiero Vincenzo LippolisEdvardas DanilaPierpaolo AlongiEdward J. PavlikPierpaolo PurpuraEdward R. SauterPierre-Aurélien BeuriatEdyta Marta BorkowskaPieter Jan SteinkampEfrosyni G. NomikouPietro AsproniEfstathios T. DetorakisPietro CerveriEfthymia ProtonotariouPietro Davide TrimarchiEfthymios BeropoulisPietro ManiscalcoEftim ZdravevskiPietro MaranoEgil NygaardPietro MazzeoEhab EssaPietro ScicchitanoEhsan AhmadpourPil Young JungEhsanul Hoque ApuPilar Alfageme-GarcíaEiichi MomotaniPim A. De JonEiichi SasakiPim PullensEiichi SatoPınar CihanEiichi WatanabePing RenEiji KiyoharaPing SongEiji MiyoshiPingjun ChenEimear McGlincheyPin-Kuei FuEisuke OzawaPiotr ArtiemjewEitaro KodaniPiotr BiałasEjay NsugbePiotr DobrowolskiEkaterina BaryshnikovaPiotr DuchnowskiEkaterina SorkinaPiotr GabryelEkram AliasPiotr GawdaEkrem SaraliogluPiotr GlinickiElaine ChowPiotr KorczynskiElaine Yee Lin ChungPiotr KulinowskiElba MaurizPiotr MalaraElena BallantePiotr Mirosław MaŁkowskiElena BernadPiotr MorasiewiczElena BertelliPiotr SkrzypczakElena CasiraghiPiotr SzczukoElena CojocaruPiotr ZapalaElena D. BazhanovaPires IvanElena DintePiyush BaindaraElena PomariPo-Cheng HsuElena PudovaPongsakorn TantilipikornElena R. LebedevaPons-Tostivint ElvireElena TchetinaPo-Ting WuElena VarottoPovilas IgnataviciusElena ZaitsevaPrabal BaruaElena-Laura AntohiPradeep Kumar DasElena-Mihaela CordeanuPradeep SinghEleonora OrtuPradeepkiran Jangampalli AdiEleonora VatamanPradipta BhaktaElham AhmadianPrakash GangadaranElia RanzatoPrakash MohanEliana Dell'olmoPrakasha KempaiahEliana GianolioPramod RangaiahEliana ScemePramod RaoElif Damla ArisanPranabananda DuttaElif NurtopPranoot TanpaiboonEline WillemsePranshu SahgalElio BignardiPrasanth BalasubramanianElisa BannonePrasanth GanesanElisa CainelliPrashant KumarElisa CairrãoPrashanth SuravajhalaElisa ReitanoPrateek BhatiaElisa ZavattaroPratiti BhadraElisabeta AntonescuPraveen Gopalakrishna PaiElisabeth ZechendorfPraveen KogantiElisabetta AntonelliPraveen Kumar BhartiElisabetta BertelliniPraveen Kumar NeeliElisabetta CanettaPraveen Kumar Reddy MaddikuntaElisabetta GroppoPravin ParajuliElisabetta MelloniPrem KushwahaElisabetta PolizziPremashis KarElisabetta StrafacePremila LeiphrakpamElisabetta VersinoPriscilla GuglielmoElise CuquemellePriyanka ChawlaElitsa Golkocheva-MarkovaPrzemyslaw ChmielewskiElizabeth C. PenickPrzemysław PłonkaElizabeth G. IngulliPrzemyslaw StrukElizabeth S. FernandesPrzemyslaw WolakElizete Aparecida LomaziPugazhenthan ThangarajuElla EvronPuo-Hsien LeEllen F. MoslethPushpa Raj JoshiElliot M. LevinePu-Ting DongElmo NeubergerQi JinEloy Martinez-HerasQi ShaoEl-sayed A. El-dahshanQi WeiEl-Sayed H. IbrahimQian DuElse Marie PinholtQian XuElvira Orduna-HospitalQiang FuElżbieta Czekajska-ChehabQiang LiuElżbieta PoniewierkaQianqian SongElzbieta RadzikowskaQichang MeiEman Abu-GharbiehQijun HuangEmanuela BarisioneQing LiEmanuela D’AngeloQing WangEmanuela LocciQinghe ZhengEmanuela TurillazziQingsu ChengEmanuele D'AmicoQingxian LuEmanuele PivettaQingyun LiuEmanuele PravatàQinyu SunEmiko Tanaka IsomuraQiu WuEmil BulatovQiuwang ZhangEmil N. SobolQuirino PiacevoliEmilie GiraudRachel CooneyEmma C. CacciolaRade M. BabićEmma D'IppolitoRadmila SparicEmma M. BentleyRadoslav BeňušEmmanouil KalampokasRadoslava KralevaEmmanouil KapetanakisRadosław GocołEmmanouil N. MagiorkinisRadoslaw JaworskiEmmanouil PikoulisRadosław KempińskiEmmanuel CognatRadosław MaksymEmmanuel J. FavaloroRadoslaw Marek KiedrowiczEmmanuel KabweRadu ChiceaEmmanuel SalawuRadu LixandroiuEmre BilginRadu TamaianEmyr BenbowRafael Camacho-CarranzaEncarna ClavijoRafael Enríquez De SalamancaEndrit ShahiniRafael LlobetEng Tat AngRafael ManzaneraEngin BerberRafael Ríos-TamayoEngy Adel AliRafael TorrejónEngy ElekhnawyRafal D. KocylowskiEnisa ShevrojaRafał GerymskiEnoch AninagyeiRafal KaminskiEnri NakayamaRafal KocylowskiEnric LimonRafał PęksaEnrico AmmiratiRafał SkowronekEnrico CarminaRaffaele FrazziEnrico FerrariRaffaele NatellaEnrico GiordanRaghavendhar R. KothaEnrico IaccinoRagnhild RøyslandEnrico MarcheseRahul ChaudhariEnrico MartinRahul K. KherEnrico SpinasRaik GrünbergEnrico StortiRaili KauppinenEnrique Alberto Guzmán-GutiérrezRaja DandamudiEnrique BoldoRajakumar RamalingamEnrique Gomez GomezRajalaxmi Rajammal RamasamyEnrique González-VergaraRajamma MathewEnrique Meléndez-HeviaRajan Kumar PandeyEnrique Pastor SellerRajani SinghEntesar DalahRajanikanth AluvaluEnwu LiuRajasree MenonEnzo Maria VingoloRajat MehrotraEphraim ParentRajeev TiwariErdenebayar UrtnasanRajesh KaluriEric A. PorschRajesh KandalaEric FelliRajinder DawraEric JeandidierRajiv SinglaEric Larry WisotzkyRajmohan DharmarajEric PeeplesRaju KhanEric RytkinRakesh Chandra JoshiEric SolaryRalf BraunEricka Noelle L'AbbéRalph G. DepalmaErik BillingRaluca Simona CostacheErik EdströmRam Kumar SelvarajuErik KudelaRamachandra BarikErik StenbergRamadhan J. MstafaErik SuRamalingam BethunaickanErika Bevilaqua RangelRaman MalathiErika CioneRamasamy AsokanErin H. SeeleyRamazan AkçanErnest U. EkpoRamgopal KashyapErnesto Di CesareRami HallacErnesto GargiuloRami S. KantarErshuai ZhangRamin E. BeyguiEsperanza Duarte-EscalanteRamin RanjbarzadehEssa M. SaiedRamiro López-MedranoEssam Al-MoraissiRamón FuentesEstefania NuñezRamon Ramon VilallongaEtienne RouxRamona D’AmicoEugen TãrcoveanuRamona Gabriela UrsuEugene KucharzRamona LeeningsEugene Tze-Chun LauRamzi MahmoudiEugenia CunhaRan DuEugénia CunhaRanieri BizzarriEugenia VlachouRanjan RamasamyEugenio MaioranoRanjeet SrivastvaEugenio MartelliRanjith PremasiriEugenio Velasco OrtegaRaphael CiumanEui-Seok LeeRaphael R. BrunoEuishin Edmund KimRaphael StrickerEunice CarrilhoRaquel De Cássia Dos SantosEuthymia VlachakiRaquel EchavarriaEva BagyinszkyRaquel LeonEva Gil-IturbeRaquel Lozano BlascoEva HasslerRasiah ThayakaranEvan CalabreseRasoul MirzaeiEvandra Strazza RodriguesRatheesh Kumar MeleppatEvangelia KoukakiRatko PrstačićEvangelia PiperiRaul Cruz-CanoEvangelos MaziotisRaúl Díaz-MolinaEvanthia BletsaRaúl Soto-CámaraEvdoxia KyriazopoulouRavesh SinghEvelio Ramirez MiquetRavi Chakra TuragaEvert Van VelsenRavi Chand BollineniEvgenia M. RapoportRavi Philip RajkumarEvgenia SitnikovaRavi Shankar ReddyEvgenii GusevRavinder KumarEvgeniy NikulchevRavinder P. KaurEvgeny E. BezsonovRavindra DhuliEvgeny KlyuchnikovRavindran Caspa GokulanEvren Önay UçarRay IlesEwa CiszkowiczRaymond SalvadorEwa KorzeniewskaRaymundo Buenrostro-MariscalEwa Toporowska-KowałskaRazique AnwerEwelina DziurkowskaRazvan DragoiEwert BengtssonRazvan SocolovEytan MorRăzvan-Ionuț PopescuEzio LanzaReda ZemaitieneEzio ZanonRedha AliEziuche Amadike UgboguRedha TaiarFabian J. BrunnerRedi RahmaniFabian SchrumpfRegent LeeFabio BaruffaldiRegina Fölster-HolstFabio Del BenReika Kawabata-IwakawaFabio Del DucaReinald BrunnerFabio Di NardoRenata ChalasFabio GuoloRenata SistoFabio ManfrediniRenata Świa̧tkowska-StodulskaFabio MendesRenato GalzioFabio PellegrinoRenaud WinzenriethFabio ZickerRené HägerlingFabrício Ramos Silvestre PereiraRené WenzlFabrizia GelardiRenjith V RaviFabrizio CedroneRens De GrootFabrizio ConsortiRenuka RamanFabrizio GentileRenzo BoldoriniFabrizio MontecuccoReuben AntonyFadi KhreishReza AbbasiFahad KhanReza ForghaniFahrettin KelestemurReza MirshahiFaidon-Marios LaskaratosReza PiriFaisal RazaReza ReiaziFalko FendRhea BhargavaFang Rong HsuRicarda HinzpeterFangfang QiaoRicardo A. Cartes‐VelásquezFangyao TangRicardo RibeiroFanpei Gloria YangRicardo Sánchez De MadariagaFariba DonovanRicardo VardascaFaridah SonsudinRicaurte Lopera-VásquezFaridoddin ShariatyRiccardo BientinesiFarzad SalehpourRiccardo MastroianniFarzad Taghizadeh-HesaryRiccardo MugliaFarzana FerdousRiccardo PascuzzoFarzaneh KordbachehRiccardo PertileFatemeh Azadegan-DehkordiRiccardo RiccardiFatemeh DabbaghRiccardo ZojaFatemeh KhatamiRichard A. AmosFatemeh ZabihollahyRichard G. TrohmanFatih HatipogluRichard J. RebelloFatma InaniciRichard John Philip BrownFausto MaffiniRichard KozarekFawzi AlbalooshiRichard LopataFederica CavalloRichard Mario PinoFederica Di SpiritoRichard OwczarzyFederica Genovesi-EbertRichard TrohmanFederica MagagnoliRie Von EybenFederica ManninoRim BourgiFederica NovazziRima HajjoFederica PaoliniRinaldo PellicanoFederica PianiRinki MinakshiFederico BrunoRita De Cássia Pontello RampazzoFederico DehoRita KissFederico HawkinsRita PavasiniFederico HerreraRitesh ThakareFederico SireciRiwa KishimotoFei GaoRizuana Iqbal HussainFei PengRizwan Ali NaqviFei YangRizwan QureshiFeifei WangRizwan WahabFeilong WangRobert AncuceanuFeipeng YangRobert AndersonFelice CrocettoRobert Andrew ShanelyFelice SiricoRobert Arthur KratzkeFeliciana Real-FernándezRobert BiedermanFelipe J. AidarRobert BreitkopfFelix BendeRobert ĆelićFelix BenningerRobert Chase CockrellFélix Javier Jiménez-JiménezRobert E. PykeFeng TangRobert GaudinFeng YangRobert KarpińskiFeng YuanRobert L. HunterFeng-Cheng LinRobert LenkinskiFengyu ChiangRobert M. HermannFengyu LiRobert NaeijeFerdinando AgrestaRobert PasławskiFerdinando FiorinoRobert RehmannFerdinando PaternostroRobert RusinaFerdows AfghahRobert Shigeo OhgamiFerhunde AysinRobert SusłoFermin García-Muñoz RodrigoRobert T. MeansFernanda Da Cruz Landim-AlvarengaRobert TroticFernando Augusto Lima MarsonRobert Walter MannFernando FonsecaRoberta ForlanoFernando LõpezRoberta GrassiFernando M. RohanRoberta GualtierottiFernando Sánchez LasherasRoberta Maria FachiniFeza DeymeerRoberta ModicaFilbert TotsinganRobertas DamaševičiusFiliberto Maria SeveriRoberto AlonsoFilip DabrowskiRoberto Bernal JaquezFilip VitoševićRoberto CampagnaFilipe Caseiro-AlvesRoberto CannataroFilipe PortelaRoberto CannellaFilippo BanchiniRoberto ChiarelliFilippo CeccatoRoberto CirocchiFilippo Crimi'Roberto FormigariFilippo FratiniRoberto GrassiFilippo MarianoRoberto LuzzatiFilippo MaselliRoberto Oliveira DantasFilippo MiglioriniRoberto PasseraFilippo PesapaneRoberto Rosas RomeroFilippos TriposkiadisRoberto ScendoniFiona AngrisanoRobin Lewis CooperFiras KobeissyRobin Singh BhadoriaFirdous A. KhanRocco CancelliereFlaminia VenaRocio Toro-CebadaFlavio BertiniRodica Maricela AnghelFlavio TarasoutchiRodolfo IulianoFleming GudaguntiRodolfo RedaFlemming AndersenRodrigo Diaz-EspinozaFlora De ContoRodrigo Martín De San AgustínFlora LiottiRodrigo ResendeFlora N. BalievaRodrigo SalasFlorence AbravanelRodrigo ValenzuelaFlorence CayetanotRodrigo Vellasco Duarte SilvestreFlorentina-Daniela MunteanuRodrigo Wladimir ValenzuelaFlorian GesslerRogelio González-GonzálezFlorian KrompRoger M. KrzyżewskiFlorian LangerRok GaspersicFlorian PoullenotRok KosirFlorian ReckerRola JaafarFlorian RenosiRoland GärtnerFlorian RosarRoland StauberFlorin George HorhatRoland ZüstFloris Van VeldenRolf SokildeFoteinos-Ioannis DimitrakopoulosRomain CoriatFotios DrakopanagiotakisRoman KotlowskiFranca FagioliRoman SmolarczykFranca NataleRomany MansourFrancesca AmbrosiRomeo MartiniFrancesca BianchiniRomualdas BausysFrancesca CarissimiRon BardinFrancesca CattoniRon KedemFrancesca De FeliceRon SaulnierFrancesca FarnetaniRonald B. BrownFrancesca GabanellaRonald Cheong Kin ChanFrancesca GranataRonald MooreFrancesca PennatiRonan MurphyFrancesca ReggianiRonan TanguyFrancesca UcchedduRong-San JiangFrancesca ZottiRonnie SebroFrancesco AsciRosa Di LiddoFrancesco BennardoRosa María Oliart-RosFrancesco BiancoRosa SorrentinoFrancesco BianconiRosalinda Guevara-GuzmánFrancesco BussuRosanna VillaniFrancesco CiodaroRosaria MeccarielloFrancesco ClapsRosario CaltabianoFrancesco D'AlòRose-Anne LavergneFrancesco Di GennaroRoshan DsouzaFrancesco Di LorenzoRoshanak GhodsFrancesco FaccioloRosita VerteramoFrancesco FerraraRossana IzzettiFrancesco FortarezzaRossella CacciolaFrancesco GallettiRossella MieleFrancesco GavelliRossella PalmaFrancesco GiammarileRossella RellaFrancesco InchingoloRoszalina RamliFrancesco MannelliRoubini ZakopoulouFrancesco PepeRoxana BohilteaFrancesco PetrellaRoxana ȘirliFrancesco SaltonRoy BeranFrancesco SalzanoRoy LauterbachFrancesco Saverio LudovichettiRóża DzierżakFrancesco Saverio SorrentinoRubaiyea FarrukeeFrancesco SecchiRubén González CrespoFrancesco SessaRuben PauwelsFrancesco SpinelliRudolf KorinthenbergFrancesco StiloRui GaoFrancesco VerdeRui VitorinoFrancisca García-Moreno NisaRui YanFrancisco Alburquerque-SendínRuitu LyuFrancisco CruzRulin ZhuangFrancisco EpeldeRundk HwaizFrancisco Espinoza-GomezRunzi SunFrancisco Javier Ávila GómezRuosen XieFrancisco Javier Povedano-MonteroRupendra ShresthaFrancisco TustumiRute SantosFranciszek BurdanRuxandra Diana SinescuFranco H. FalconeRyan AccordFrancois CornelisRyan McGrathFrançois E. J. A. WillemssenRymantė GleiznienėFrancois EyskensRyo NakamaruFrançois JamarRyogo MinamimotoFrancois KiemdeRyong Nam KimFrançois PetitpierreRyosuke TonozukaFrançois PhilippartRyszard PlutaFrançoise Galateau SalleRytis MaskeliūnasFrane PaicS. M. Yasir ArafatFrank A. SchildbergS. N. DeepaFrank Mayta-TovalinoSaad HussainFrank N.M. TwiskSaad Ihsan ButtFrank TraubSaba BattelinoFrank Vinholt SchiødtSabina CapellariFrank WeberSabine LehFrantz Rom PoulsenSabrina LisiFranz Felix KonenSabrina MartinezFranz WegnerSabrina TosiFrauke StankeSachi Nandan MohantyFred CohenSachio TakenoFreddy A. LucaySachit AnandFrederick H SilverSadettin CiftciFrederick W. DamenSadiq HussainFrederik DenormeSadiya ParveenFrederique RetornazSae-Bae NapaFredrik SchjesvoldSaeed AnwarFrikkie J.W. CalitzSaeed M. BamatrafFu-An LiSafwan OmranFuhito HojoSagar PandeFulya OzerSajjad ShiraziFumitaka KogaSakib K PathanFutian WengSakineh HajebrahimiFu-Zong WuSakthisudhan KaruppananG. MaragathamSalama MostafaaGábor KonczSalavat AglyamovGábor SomlyaiSalem Youssef MohamedGabriel Claudiu TenderSalim Si-MohamedGabriel Dias RodriguesSalvador PérezGabriel NallathambiSalvatore BocchieriGabriela Anaya-SaavedraSalvatore LeonardiGabriela AniteiSalvatore SciacchitanoGabriela GoujgoulovaSalvatore SorrentiGabriela-Dumitrita StanciuSalvatore TedescoGabriele GhettiSamar TharwatGabriele SchoenianSamara Tatielle Monteiro GomesGabriella CsíkSambit MishraGabriella SerioSameena PathanGaetano GalloSami RamadanGaetano MarvertiSamir BarghoutGaetano PaoneSampath Dakshina Murthy AchantaGaetano ReaSamrad MehrabiGaetano SantulliSamrita DograGaetano ScaramuzzoSamuel PushparajGagandeep SagguSanda Maria CrețoiuGagliardi Anna RobertaSandeep Kumar ChavanGalia Ramírez-TolozaSandeep Kumar MishraGalina IlievaSandi Baressi ŠegotaGalina ShepelkovaSandip N. ChavanGalliera Emanuela RitaSandra DiederichGang YeSandra HirschGannon C. K. MakSandra LeiszGary D. SteinbergSandra MilićGary L. NormanSandra P. NunesGaryfallia PeperaSandra RebeloGąsowska-Bajger BeataSandra TrapaniGaurav JoshiSandro GlumacGautam SethiSang Hoon KimGayathri Thevi SelvarajahSang Jun LeeGebhard MathisSang Moo LimGeeta Devi LeishangthemSang Tae ChoiGemma RossiSangsoo KimGennaro VessioSang-Woon KimGeok Chin TanSanika SuvarnapathakiGeorg SingerSanja Lovric KojundzicGeorge A. PapakostasSanjeev B. KhanagarGeorge Alexandru MafteiSanjeev KhanagarGeorge FotakopoulosSanthosh ChidangilGeorge L. HinesSantiago CepedaGeorge MandyburSantosh GeorgeGeorge Mihail VlăsceanuSara BoccaliniGeorge NotasSara BozziniGeorge S. KorresSara C. ZapicoGeorge SamanidisSara CostanzoGeorge ShirreffSara Danzi-EngoronGeorge TavartkiladzeSara E. HockerGeorgia KarpathiouSara FranziGeorgios A. PappasSara GaspariniGeorgios BenetosSara RicciardiGeorgios D. MichailSarah AdamoGeorgios IatrakisSarah Kittel-SchneiderGeorgios KatsarasSarah MattonenGeorgios LaliotisSarassunta UcciGeorgios LeontidisSarhang HamaGeorgios ManikisSarinder Kaur Kashmir SinghGeorgios VrakasSaritphat OrrapinGe-Peng JiSarv PriyaGerald J. KostSaša ČučnikGerald SeinostSasa RajsicGerard H. JansenSastre-Garau XavierGerard M. MoloneySathya Baarathi Shanthi RavichandranGerard R. AverySatilmis BilginGerardo CazzatoSatoru HiwaGerardo PellegrinoSatoru MunakataGerasimos BaltsaviasSatoshi KobayashiGerasimos EvangelatosSatoshi TakahashiGereon SchälteSatoshi YuguchiGergely FeherSatya Eswari JujjavarapuGerman A. ArenasSaud AlrawailiGerman PerezSaúl Martín RodríguezGhasem SolgiSaul Tovar-ArriagaGhassan BkailySaulius JuodkazisGheorghe Nicusor PopSaulius SvagzdysGhulam MuhammadSaurabh LuthraGhulam MuhiuddinSaverio CandidoGiacomo CasaliniSaverio MuscoliGiacomo DomeniciSavina MannarinoGiacomo RossittoSavvas LampridisGiacomo TondoSawan JalnapurkarGiada BianchettiSayan SarkarGiada Maria VecchioSayantani ChowdhuryGiamberto CasiniSayed Ameenuddin IrfanGiampietro FarronatoSayed Sartaj SohrabGiampietro GubbiniScott CoussensGian Franco ZannoniSebastiaan Van SteenselGian Luca MarellaSebastian D. SchaeferGian Luca Rampioni VinciguerraSebastian IwaszenkoGian Luigi NicolosiSebastian MertowskiGian Marco GiuseppettiSebastian NagelGian Paolo CavigliaSebastian O. DeckerGiancarlo GarutiSebastian OnciulGianCarlo PecorariSebastian SchollGiancarlo ScognamiglioSebastian TschaunerGiandomenico MaggioreSebastian WawrockiGiane Regina PaludoSebastiano A. G. LavaGianluca CasseseSebastiano CiccoGianluca FaddaSébastien SchmerberGianluca GennarelliSedami GnidehouGianluca LuccheseSeema KumariGianluca Raffaello DamianiSefer ElezkurtajGianluca SmerilliSeham El-KassasGianluigi PastaSehar IqbalGianmaria SalvioSeil KimGianna DiPalmaSeisuke OkazawaGiannis D. ParaskevopoulosSelami Aykut TemizGianpaolo RossiSelena Y. LinGiau Van VoSe-Lim OhGiedrius BarauskasSelma DemirGilles LesieurSemra BilaçeroǧluGina GheorgheSengul DoganGiner Galván VicenteSenija EminovićGino GrifoniŞenol TonyalıGioele GavazziSenthilkumar SankararamanGiorgia FioriSeo Hee YoonGiorgio BoganiSeong Beom ChoGiorgio CalloviniSeounman YuGiorgio GallinellaSepideh N. AsadbeigiGiorgio LofreseSeppo Wang LangerGiorgio TulliSerafeim C. ChaintoutisGiou-Teng YiangSerbülent TürkGiovanna FerraioliSerena Dell'AversanaGiovanna GalloSerena GasperiniGiovanna GiordanoSerena StiglianoGiovanna MantiniSergey BrodskyGiovanna RussoSergey GurevichGiovanni BarassiSergey MaksimovGiovanni Battista Levi SandriSergey Pavlovich OsipovGiovanni BocciaSerghei CovantevGiovanni ButturiniSergi Vidal-SicartGiovanni CimminoSergio CortelazzoGiovanni CristalliSergio Crespo-GarciaGiovanni DamianiSergio García-GarcíaGiovanni DiracoSergio HaimovichGiovanni Donato AquaroSergio NavarroGiovanni E. GiganteSergio Rius-PérezGiovanni MaccioniSergiu FendrihanGiovanni MirabellaSerkan YílmazGiovanni MuscasSeung Uk LeeGiovanni PalleschiSeunggyun HaGiovanni PaolinoSeung-Hun LeeGiovanni ScambiaSeverin RodlerGiovanni TazzioliSeverino PersechinoGiovanni TossettaSeyed Ahmad Naseri-AlaviGiovanni TrisolinoSeyed Mohammad AyyoubzadehGirardelli SerenaSeyed Mohammad Kazem AghamirGiseli KlassenSeyedmehdi Mehdi D. PayabvashGisella VischiniSeyit Ahmet ErolGiulia D'AmatiShabana Amanda AliGiulia Elena MandoliShabana HabibGiulia FerrarazzoShadi H. TabibianGiulia FestaShafiq Ur RahmanGiulia MorsicaShahanshah KhanGiulia SantoShahid MansuriGiulia SciosciaShailender SinghGiulia TrovarelliShailendra SinghGiulia ZamboniShajahan AnverGiulio ArcangeliShamir CassimGiulio FrancoliniShamroop Kumar MallelaGiulio RossiShams TabrezGiuseppe AcriShan MouGiuseppe BarisanoShane FoleyGiuseppe BattagliaShangrong FanGiuseppe BiaginiShankargouda PatilGiuseppe BroggiShanshan TanGiuseppe CalabreseShantanu GuptaGiuseppe CastaldoShaobin WangGiuseppe CoratellaShaofeng YanGiuseppe De VincentisShaopeng PeiGiuseppe Di CaprioShaoqiu HeGiuseppe Di MartinoSharmila FagooneeGiuseppe Emmanuele UmanaSharon K. MichelhaughGiuseppe Fabio ParisiSharon Tirosh-LevyGiuseppe FanettiShaun SabicoGiuseppe G. LoscoccoShazid Md. SharkerGiuseppe GulloShebli AtrashGiuseppe IatíSheeba Jenifer SujitGiuseppe JurmanSheeba SujitGiuseppe LiberatoreSheeja Saji VargheseGiuseppe Lo GiudiceSheikh Mohammad Fazle AkbarGiuseppe LosurdoSheikh Shanawaz MostafaGiuseppe MaccagnanoSheng FuGiuseppe MagliuloSheng Wen Steven ShawGiuseppe MasciaSheng-Chieh ChanGiuseppe MaulucciSheng-Dean LuoGiuseppe MinerviniSheng-Hung ChenGiuseppe MorgiaSheng-Lih YehGiuseppe MurdacaShianshiang WangGiuseppe RicciShibdas BanerjeeGiuseppe RivaShichao LvGiuseppe SolarinoShigao HuangGiuseppe SprianoShigehiro KarashimaGladys KoShigeki MatsubaraGlenn Van SteenkisteShigenaga MatsuiGong ZhangShigeo IshikawaGoo-Rak KwonShigeo TakebayashiGopal Kumar PatidarShigeo YamamuraGopi BattineniShihlung ChenGoran AugustinShihori TanabeGoran VujićShih-Yen LoGorazd PojeShih-Yi LinGordana Wozniak-KnoppShilpi AgrawalGorkem MemisogluShingo TsujinakaGour GoswamiShingo YamamotoGraça PortoShin-Hoo ParkGraciela Caire JuveraShinichi OhtaGrady NelsonShin-Ichi OsadaGrazia BossiShinji TakamatsuGrazia D'OnofrioShinji TeramotoGrazia PaviaShin-Ming HuangGraziella BonettiShinnkuang LinGrażyna Wyszyńska-PawelecShintaro NakanoGrégoire CattanShintaro SukegawaGregor PoglajenShinya MatsuzakiGregor ProsenShipra AgarwalGregor StavrouShirisha JonnalagaddaGregorio BernabéShiro UrayamaGregorius Satia BudhiShitala PrasadGregory ConnersShiu-Feng HuangGregory P. BartonShiv VermaGregory W. AlbertShixiong ZhangGrigorios TsigkasShoichiro TangeGrigoris AmoutziasShoji OuraGrissell TiradoShoko HaraGrzegorz CieslarShooka MohammadiGrzegorz JakubiakShubham MahajanGrzegorz RabaShuji MomoseGrzegorz TrybekShuji OzakiGrzegorz ZielińskiShujiro HayashiGsrsnk NaiduShu-Jui KuoGuangyao WuShuko NojiriGuanqiu QiShumin YangGuanyu PiaoShumit SahaGudrun M. FeuchtnerShuncong WangGuennadi SaikoShun-Fa YangGuglielmo Actis DatoShunsuke FurutaniGuglielmo ManticaShunsuke SawadaGuglielmo Niccolò PiozziShuntai ZhouGuglielmo RamieriShuoming OuGuido Alberto Massimo TiberioShuo-Tsung ChenGuido CaluoriShuowei CaiGuido CostaShweta TikooGuido FitzeShwu Jiuan SheuGuido PellettiSiddharth MatikondaGuido ValenteSiddhartha BhattacharyaGuigen LiuSiegbert FaissGuillermo A. Cañadas-De La FuenteSiham Chafai ElalaouiGuillermo LaheraSilke Anna Theresa WeberGuillermo Romero-FarinaSilvano DragonieriGuillermo RusSilvano FerriniGuillermo Til-PerezSilverio RotondiGulzhanat AimagambetovaSilvia Alejandra García-GascaGuna LaganovskaSilvia CapuaniGunavathi C.Silvia García-BujalanceGundula Gesine Schulze-TanzilSilvia GiovanniniGündüz YümünSilvia IppolitoGunnar L. OlssonSilvia NovíoGüntülü AkSilvia PalmaGuo Dong GaoSilvia Stefania LongoniGuofang XuSilvia SvegliatiGuofang ZhangSilvia TortorelliGuolin MaSílvio César CazellaGuozheng WangSimge Taşar FarukGurmukteshwar SinghSimon D. BamforthGurudeeban SelvarajSimon SchoechlinGustavo FernandesSimon T. AbramsGuy FroyenSimon VulfsonsGuy R. AdamiSimona Delle MonacheGwang Jun KimSimona Di MeoGwenaël PagéSimona IoaniţescuGyan ChandSimona ManoleGyörgy SzabóSimona MartinottiHabib HamamSimona MoldovanuHabib KhanSimona RollaHacı SaglamSimona Roxana GeorgescuHadi JumaatSimone AppenzellerHadith RastadSimone BelliHady HaririanSimone ConciHaewon ByeonSimone GrassiHafiz Tayyab RaufSimone MorraHagen FrickmannSimone MorselliHaiming LuoSimone NataliHaipeng LiuSimone PernaHaipeng XiaoSimone SerafiniHaiyun LiSimone VanoniHajime TakatoriSimone WajnerHamed JelodarŠimons ŠvirskisHamed NosratiSina RashediHamid NasiriSinan DenizHamid OsmanSitthichok ChaichuleeHamideh KerdegariSiu Wai ChoiHamideh SalehiSiva Prasad PandaHamzeh GhorbaniSivanesan DakshanamurthyHanaa SalemSivapong SungpraditHanane AlliouiSivaraman NatarajanHaneen B. AliSławomir Cezary ZmonarskiHannah C. AinsworthSławomir KujawskiHannah M. ThomasSławomir WołczyńskiHans BrunnströmSławomir ZmonarskiHans ChristiansenSlobodanka Lemajic-KomazecHans G. DrexlerSmita NairHans-Christian SiebertSmriti PandaHansda ShekharSneha ShahHan-Sheng ChuangSneha SitaramanHan-sin JeongSnekhalatha UmapathyHans-Jürgen BiersackSofia Barbosa-GouveiaHanwen WangSofia PillaiHany S. HusseinSofoklis MitsosHaobijam BasantaSohail KhalidHaomin LiSohail SyedHaoyin ZhouSohel M. JuloviHarald MatthesSoichiro MurataHariharan MuthusamySokou RozetaHarikrishnan R.Somashekar G. KrishnaHarikumar RajaguruSomchai AmornyotinHarinder GillSomdutt MujwarHarry Victor Michael SpiersSomenath ChakrabortyHartmuth NowakSonam KumariHaruka AbeSonam MittalHasan SymumSonay AydınHassan AlbarqiSong YangHassan Mohammad NaifSongli ZhuHasti Kamali SarvestaniSönke LangnerHaytham ElgharablySonu M. BhaskarHazrat AliSonu Menachem Maimonides BhaskarHe LiSoonchul LeeHeather WilkinsSooyeol LeeHeba AhmedSophie Le CannHedelin HenrikSophie MavrogeniHeejin KimSoraia MeloHeike ReinhardtSoraya El GhannudiHeitor MorenoSorin BaracHélder CardosoSorin HostiucHelen FogartySorin OlariuHelena FelgueirasSotiria Dimitrios VrouvaHelena NavasSotirios KakavasHelmut DittmannSotirios SotiriouHelmut H. TelleSotiris KotsiantisHelmut MairSougata GhoshHeming YaoSoumaya KouidhiHenk SchalligSoundappan S. V. SoundappanHenk SchonewilleSourish GhoshHenning HagmannSpela Stunf PuklHenriette HeinrichSpyridon KintziosHenrik C. BäckerSricharan S. VeeturiHenrique SilvaSrikanth PrabhuHenry SutantoSrinivas AluriHenryk MazurekSrinivas SistlaHerbert LoellgenSrinivasa Reddy BonamHerbert Michael HeiseSriram SundaravelHermann GillySrjit DasHervé ReychlerStåle NygårdHesam BabahosseiniStamatia TheodoridouHiam AlquranStamatoula TsikrikaHideaki KawabataStanislav PantelyushinHideaki OkajimaStanislav RubakhinHidehiko OkazawaStanislaw DejaHidehiko TakigawaStefan AcostaHideki KobaraStefan HohausHideki MiyachiStefan NaydenovHideo HashizumeStefan Paul-AndreiHideo KatoStefan PszczolkowskiHideo WadaStefan WesargHideto ImuraStefan WinterHidetoshi NojiriStefania CimbanassiHideyuki KinoshitaStefania EspaHimanshi AggarwalStefania MagdaHinhin KoStefanie WillekensHira FatimaȘtefan-Ioan StratulHirawati DevalStefano AlbaniHiroaki KonishiStefano CarugoHiroaki MiyoshiStefano EramoHiroaki NekiStefano Francesco CrinòHiroki TeragawaStefano GhioHiroki YokotaStefano Giannoni-LuzaHiromi KurokawaStefano Marco Paolo RossiHiromichi YumotoStefano MarroneHironari TamiyaStefano MenzoHiroshi InuiStefano NistriHiroshi KeinoStefano PalermiHiroshi KurokiStefano PileriHiroshi MiyamotoStefano RattiHirotomo KatoStefano RealdonHiroyoshi MoriStefano SivolellaHiroyuki KunishimaSteffen HauptmannHiroyuki TakeiSteffen RosahlHiroyuki YamadaSteffi M. UrbschatHisako FujiharaStella CascioferroHo TsoiSten HellströmHo-Fung ChanStepan FeduniwHoi Leong Xavier WongŠtěpán HavránekHoi-Ying Elsie YuStephan Alexander BraunHojong ChoiStephan AltmayerHolger A. LindnerStephanie A. Lee-FelkerHolger H. SiguschStephanie A. ThomovskyHolger LoessnerStephanie SnowHollis O'NealStephanie SteidlerHong DuanStephen A. ZdericHong HuangStephen Chun Lun LauHong XuStephen R. KlapperHong-Gam LeStephen RichardsHonglei ChenStephen S. PalmerHongliang HeStephen YipHongnan CaoStergios BoussiosHongping JinSteve JefferyHongyu WangSteven LevyHong-Zen YehSu Hwan KimHongzhu CuiSu Kang KimHon-Yi ShiSu Keng TanHooman EsfandiariSubbaramiah SridharHoraţiu MoldovanSubhadip ChoudhuriHorea FeierSubrato BharatiHoria StancaSudeep TanwarHossam Magdy BalahaSuganthaPriya ElayapillaiHossein Mohammad-RahimiSugato HajraHossein MozdaraniSuhail AkhtarHouda HarkatSuhwan LeeHouman SotoudehSuhyun ParkHrayr AttarianSuhyung ParkHrvoje BudinčevićSukanta SabutHsiang-Ning LukSukhvinder SinghHsiao-Hsuan KuoSuk-Ja YoonHsin-Yao WangŞükrü ÇağlarHsueh-Ju LuSumadi Lukman AnwarHsu-heng YenSuman ChaudharyHuafeng WangSuman WattalHuaifu DengSumit Randhir SinghHuang-Pin WuSunayana Begum SyedHuan-Wu ChenSuneel KumarHuapeng FengSung Eun KimHua-Sheng ChiuSung Ki LeeHubertus AxerSung-Hoon MoonHugh DevlinSunghun NaHugo Martínez-RojanoSung-Jig LimHugo Pena-VerdealSung-Lang ChenHugo RochaSung-Soo KimHui HuangSung-Tae HongHui-Ching ChuangSunil JaimanHuifen ZhouSunil KarhadkarHui-Hua HsiaoSunil Kumar SansaniwalHui-Ju LinSunil Kumar SinghHuiling ChenSunita GhoshHui-Yen ChuangSunny Chi Lik AuHumberto DebatSunny RajHung-Wei WangSuparshya Babu SukhavasiHungwon TchahSupriya SharmaHun-Ju YuSupriyo RayHuong Carisa Le-PetrossSurachai Sae-JungHurng-Yi WangSurasak KasetsirikulHuseyin KusetogullariSuravajhala PrashanthHussain Mohammed Dipu KabirSuresh KalathilHwa-Jin ChoSurya KantHye Chang RhimSusan A. FarrHye-Geum KimSusan MollanHye-Jin YouSusana LechugaHyo-Young LeeSusana R. SousaHyun Soo KimSusanne RospleszczHyung-Seok KimSusmita BarmanHyung-Youl ParkSusumu MatsukumaHyun-Jin MinSuzuki ShugoHyunjoong KimSven FlemmingHyunyeol LeeSven Van IjzendoornI. Jung TsaiSvetlana AnticIan Kim B. TabiosSwapnil SanmukhIbrahim M. ChamseddineSwarnalatha RajaguruIbrahim M. SayedSwati AnandIchiro YamadaSwati JainIdan GorenSwinburne AugustineIgnacio Ruz-CaracuelSyed Ahmad Chan BukhariIgor A. ZolotukhinSyed AhmedIgor Age KosSyed Furqan QadriIgor DumicSyed Haris OmarIgor KhatkovSyed NabilIgor OscorbinSyed RahmanuddinIgor P. KaidashevSyoichi TashiroIgor PiotrowskiTadahisa InoueIkkei TamadaTadashi ItoIkram SghaierTadashi NakamuraIkuya MiyamotoTadatsugu MorimotoIlan MerdlerTadayuki TakagiIlaria GrassiTadeusz PrzybyłowskiIlaria MarcantoniTae Hoon RohIldiko SzantoTae Keun YooIleana ConstantinescuTae-Yoon LeeIlias P. NikasTafuri AlessandroIlke Coskun BenlidayiTahira NazirIlkka K. IlonenTahmina Nasrin PolyIlona DjoricTai-Ho ChenIlya KirgnerTai-Hua YangIlya UlasovTaimur HassanIlyes BenlalaTairo KuritaIl-Youp KwakTajana Stoos-VeicIlze ŠtrumfaTakaaki SenbonmatsuIman KhodarhmiTakaaki TsuchidaImena RexhepiTakahiro EinamaImtiyaz Ahmad WaniTakahiro HiraideIn Koo HwangTakahito NakajimaIna Valeria ZurloTakashi HimotoInchang HwangTakashi MurakamiInes KožuhTakashi NiizekiInes P. MarquesTakashi OgawaInês SantosTakashi OnoIngrid MartonTakashi SatoIngrid TonniTakashi TanakaIn-Lu Amy LiuTakayoshi ShinyaInmaculada Barranco-ChamorroTakayuki TakedaInnocent AfekeTakefumi KimuraIoan SporeaTakeji Takamura-EnyaIoan TileaTakemi OtsukiIoana CabaTakemichi FukasawaIoana MartuTakenori InomataIoana MozosTakeshi HiuIoanina ParlatescuTakeshi KannoIoannis A. GiantsisTakeshi NishidaIoannis ApostolopoulosTaku KouroIoannis BoutasTakuji TanakaIoannis K. ToumpoulisTakuma InagawaIoannis KonstantinidisTakuya KoieIoannis Ntanasis-StathopoulosTalha Burak ALAKUŞIoannis PetrakisTalha Mahboob AlamIoannis RallisTamara ČačevIoannis VamvakarisTamara GulicIolanda Valentina PopaTamás BaranyaiIon Alexandru BobulescuTamás HaideggerIonut LuchianTami Brutman-BarazaniIonut TudoranceaTami LivnatIosif MarincuTânia Soares MartinsIpsita MohantyTanuj PuriIqbal QasimTanushree DangiIraklis TsangarisTanya StratevaIrena HocevarTanyalak ParimonIrena IlicTao HuangIrena JekovaTao YangIrena Seili-BekafigoTar Choon AwIrena TabainTarek ElshebinyIrena Wojsyk – BanaszakTareq KhanIrene BurckhardtTariq AliIrene EspositoTarja SironenIrene KaranasiouTarr TündeIrene MadrigalTaru SinghIrfan MohamadTasadduq ImamIrina Iuliana CostacheTasneem MotiwalaIrina MateiTatjana RadosavljevićIrina MizevaTatsat PatelIrina MocanuTatsuo KandaIrina TicaTatsuya ImabayashiIrma Gonzalez–CurielTatsuya ToyokawaIrmina MorawskaTatu RojalinIrshad AhmadTatyana BaltinaIryna RusanovaTavan JanvilisriIryna SamarskaTaye Girma DebeleeIsabel Cláudia Masson Poiares BaptistaTeddy CraciunescuIsabel ColmeneroTena L. RosserIsabel Corrales GutiérrezTerence Chi Chun TamIsabel Escobio-PrietoTeresa PerraIsabel Faria-RamosTereza GrimmichováIsabel LarreTerrence PivaIsabel Poiares BaptistaTerry Cheuk-Fung YipIsabel SassoonTeru KamogashiraIsabella AquilaTeruhiko ImamuraIsabelle AyxTeruya KomatsuIsah A. LawalTetiana HovorushchenkoIsain ZapataTetsuo NishikawaIsmaheel LawalTetsuya IshikawaIsmaheel O. LawalTetsuya TakahashiIsmini PapageorgiouTetsuya TakikawaIsrael NissanThadeu BritoIssa AtoumThakshila LiyanageIssanov AlpamysThanh-Canh HuynhIstván PetákThanongchai SiriapisithIta HadžisejdićTheo M. De ReijkeIulen Cabeza-GilTheodoros FouskasIulia-Cristina BagiuTheodoros KarantanosIulian AntoniacTheofani AntoniouIuliana NenuTheofilos M. KolettisIvan BrandslundTheoni KanellopoulouIvan BruknerThi Thao Linh NguyenIvan ČekerevacThijs Van Der ZijdenIvan IzoninThirumal RajIvan MalčićThomas BaumIvan MiskulinThomas BurgoyneIvan PalaginThomas D. GossiosIvan SabolThomas Franz KrebsIvan ŠošaThomas FrauenfelderIván Valdivia-GandurThomas GeyerIvan VellaThomas J. GastIvana JarakThomas J. T. P. Van Den BergIvana KholováThomas KallarakkalIvana P. NedeljkovicThomas M. TiefenboeckIvana SamarzijaThomas RensonIvana TrivicThomas SartorettiIveta SelingerovaThomas SchaibleIvo DraganovThomas SenonerIvo Dumic-CuleThomas StammingerIwona PuzioThomas W. WainwrightIwona RybakowskaThomas WolberIzabela RaganThomas WolfIzabella Karska‐BastaThomas ZellersJ. Andrew OnesimuTiago RibeiroJ. Matthew HinkleyTiago Severo PeixeJacek GagalaTianfei ZhouJacky LooTiantian YangJacobo Rodríguez-SanzTianyu ZhengJacobus Van Der VeldenTianyun ZhangJacopo NoriTiberiu Paul NeaguJacques GilloteauxTibor GuzsvineczJae Ho LeeTie-Qiang LiJae Yon WonTiffany Champagne-LangabeerJae-Hyun KwonTijana Išić DenčićJae-Myung RyuTilili BarhoumiJae-Woo LeeTill BraunschweigJae-Yong KimTilman SteinertJafar RezaieTim PrangemeierJahanzeb MalikTim WalkerJahnavi AluriTimothy John MahonyJaime A. RinconTimothy KwaJaime Bustos-MartínezTimothy RittmanJaime DevineTina Tičinović KurirJainil ShahTing ZhongJair CervantesTinhinane FaliJakob GubensekTin-Yu WuJakub KufelTissa WijeratneJakub NalepaTizian RosenstockJakub ZakTobias PuengelJalal TaneeraTobias TodsenJames Bowen StiehlTolga CukurJames ChowTolga ÖzmenJames Chung Wai CheungTomás Arias-VergaraJames CovingtonTomáš BüchlerJames DoubTomas NovotnyJames GristTomas SimurdaJames Janopaul-NaylorTomasz BartkowiakJames Kit-hon TsoiTomasz FrączykJames Lee CraineyTomasz FuchsJames PowersTomasz K. NowickiJames SolomonTomasz KulczykJamir Pitton RissardoTomasz MarjanskiJamshid Abdul-GhafarTomasz OrczykJan BilskiTomasz RymarczykJan Darius UnterlauftTomasz TokarekJan DommerholtTomasz UrbanowiczJan EggerTomasz WolnyJan Irma PoelaertTomasz ZielinskiJan KotarskiTomica HrenarJán KozempelTomislav KuzmanJan KühnischTomislav MeštrovićJan Maciej ZauchaTomislav VladušićJan MalikTommaso BocciJan TrnaTommaso ErcoliJan Van Den StockTommaso NeriJan Van SchaikTommaso SteccaJan VrbaTomoaki MiyagawaJan ZabrzyńskiTomohiro YazawaJana SmahelovaTomoki NakamuraJane FletcherTomotaka SaitoJane K. NguyenTomoya KobayashiJaneen R. JordanTomoya MaedaJanewit WongboonsinTomoya TakabayashiJang Gyu ChaTomoyuki KoikeJanggun JoToru KamedaJangwoo LeeToru KannoJanka BábíčkováToshiharu FujiiJanna MorawitzToshihide TanakaJanne MäkeläToshihiko TashimaJanusz SielskiToshihiro NankiJan-Willem BeenakkerToshihiro TsurudaJaromir AstlToshimi ChibaJaroslav ČermákToshinungla AoJarosław PasekToshio IchikiJaroslaw RegułaToshiyuki FurukawaJarosław TrȩbaczToshiyuki MatsuiJarosław ZubrzyckiTovah CowanJasbir UpadhyayaTraian DumitraşcuJasmina Casals TerreTrevor ArcherJasmine Pramila DavadhasanTrevor BraselJason J. WangTri Niswati UtamiJason X. ChengTriantafyllos DidangelosJasti SateeshTrieu NguyenJaume Coll-FontTrifan Anca-VictoritaJavad AlizargarTristan T. HormelJavad JavidniaTrung Duy TranJaved Mehedi ShamratTsuguru HayashiJaveed ShaikTsuguto FujimotoJavier Francisco-MorcilloTsung-Cheng YinJavier Ramos-OrtegaTsung-Hsien LeeJaw-Yuan WangTsung-li LinJay M. StewartTsuyoshi ShiraiJayachandran VenkatesanTsuyoshi TajikaJayesh J. AhireTsvetelina VelikovaJayson StoffmanTuba Çiğdem OğuzoğluJean Christophe DelpechTuba YilmazJean Christophe IanottoTudor Sorin PopJean Jacques MonsuezTu-Hsuan ChangJean VanderpasTurki TurkiJean-Baptiste RiccoTvrtko HudolinJeanette KoeppeTyng Yuan JangJean-Francois Lauzon-JosetTyrone PretoriusJean-Jacques MonsuezTze-Chien ChenJean-Marc BarretTzu-Chuan HoJeanne Wilson-RawlsUdaiyappan JanakiramanJean-Paul MakhzoumUdo RolleJean‐Paul MarieUgo CarraroJean-Paul MottaUiju ChoJee-Young ParkUjjal Kumar BhawalJeffrey HordUlf GunnarssonJeffrey NeashamUlf SchminkeJehad AliUlf TitzeJeih-Jang LiouUlrike BacherJelena KovacUma DebiJelena MilasinUmaima Al-alemJelena Nesovic OstojicUmair KhanJelica Grujić-MilanovićUmberto GaragiolaJelle OverboschUmberto MalapelleJemma KernsUmesh Chandra BeheraJenchih TsengUmut VarolJenette CreaneyUpendra RathoreJennifer HowcroftUriel ZapataJennifer R. Stapleton-KotloskiUrsula PanznerJennifer SalinasUrsula WindbergerJenn-Ming YangUrszula MalinowskaJen-Pin ChuangUsman MuhammadJens André HammerlUtkarsh KohliJens KirchnerUzair IqbalJens-Peter KühnUzair SajjadJeong Sook KimUzma SamadaniJeong Won LeeV. ArulkumarJeong-An GimVadim ElaginJeong-Hoon LeeVadim V. GenkelJeremias Bayon LorenzoVahid AbolghasemiJeremie D. OliverVaideesh ParasaramJérémie PrévostVajira Lasantha Bandara ThambawitaJerome DelhommelleValentina DiniJérôme TorrisaniValentina Elisabetta BounousJerónimo González-BernalValentina GentiliJerzy ChudekValentina PileczkiJerzy ŚwiątekValentina PinelliJerzy WalochaValentina VassilenkoJesse R. QualliotineValentina VicennatiJessica CoertseValeria CuccariniJessica IorioValeria IsellaJessica L. GiffordValeria SebriJessica RossiValérie ChoumetJesus Antonio Gonzalez-Hermosillo GonzalezValérie GaudinJesús Jiménez-LópezValerio Di PaolaJesús Molina-MulaValerio D'OraziJesus Oliva Pascual-VacaValerio Francesco AnneseJi Hyun KimValerio Gaetano VelloneJiafen HuValerio LeoniJiahao PengValerio NardoneJialei DuanValeriu Marin ȘurlinJia-Ling RuanValeriy AndreevJian WangValery V. LikhvantsevJianbin LiValliappan RamanJiang LiValner BrusamarelloJiangang ChenVan-An DuongJianjun WenVance Girard NielsenJiann-Her LinVanessa De PaceJianping LuVanessa Stadlbauer-KöllnerJianqiang XuVarsha PotdarJianqiu KongVartika TomarJianwei ShuaiVarun GuptaJian-Xing WuVarun KumarJiaqi ZhangVasco PoncianoJiawei WangVasile DrugJiaxing ZhangVasileios PapatsirosJie DingVasiliki N. IkonomidouJie JiaVasilios PerifanisJie SunVasilisa V KrasitskayaJie TianVasily KashtalapJien-Jiun ChenVassilis GeorgouliasJiheum PaekVassilis KoutoulidisJihye LimVassilis L. TzounakasJill TippingVeeranoot NissapatornJim ParkerVelia D'AgataJim SheuVenkatesan RajinkanthJin BaiVenkatesh AvulaJin WangVenkatesh RangarajanJin WeiVera CelicJin Yong JeongVerena Ahlgrimm-SiessJin YuanVerena DamianiJing GaoVeronica MacchiJing GuoVeronika HuntosovaJing TianVeronika HuntošováJing XuVéronique KemmelJing YuanVesa Cosmin MihaiJinglin ZhangVesna I. KesićJingping WangVessela KrastevaJingxia LiuVibhor KumarJingyi ChiVibhu Krishnan ViswanathanJingyi ZhaoVibor MilunovićJingyu WangVicenç LeónJin-Hwa JungVictor FloresJinjun XiaVictor KallenJinming HuVíctor M. CampelloJinn-Rung KuoVictor Manuel Mendoza-NuñezJinshan TangVictor MartinJinxiang XiVictor W. WeednJinzhou ZhuVidya RattanJiong MeiViet TranJitendra KumarVijaya Anand ArumugamJitendra Kumar TripathiVijayakumar SukumaranJiuann-Huey Ivy LinViktor ChrobokJiwan GeVille LeinonenJiwon BaekVinayakumar RaviJo Anne H. YoungVincent DochezJo KatoVincent PonsJoana VitalléVincent RibragJoan-Lluis Vives-CorronsVincentas VeikutisJoanna DąbrowskaVincenza CarchioloJoanna GołębiewskaVincenza GianfrediJoanna LudzikVincenzo CastiglioneJoanna SikoraVincenzo FiorentinoJoanna SzczepanekVincenzo NuzziJoanna TrelinskaVincenzo PositanoJoanna ZawitkowskaVincenzo VillanacciJoão Gama MarquesVinit BaliyanJoão Gaspar-MarquesVinod KumarJoão Marcelo TeixeiraVioletta Opoka-WiniarskaJoão MendesVirupakshappa VirupakshappaJoão Pinto-De-SousaVishal ChavdaJoão SantosVishal G. ShelatJoão-Paulo-Mendes TribstVishalkumar G ShelatJoaquim CarrerasVishu MishraJoaquim PinheiroViswas Raja SolomonJoaquin Gayoso CabadaVít HerynekJoaquin Manzo-MerinoVitalii PavlovJoaquín María CamposVitaly I. KoberJochen MaulVitaly VolpertJochum WolframVito Andrea CapozziJoerg-Ingolf SteinVito LongoJohan A. WesterhuisVittoria PerrottiJohanna S. UngerstedtVittorio AbbonanteJohannes Dominikus PalluaVittorio AprileJohannes HammelVittorio CapuanoJohannes HatzlVittorio ChecchiJohannes LubkeVittorio FineschiJohannes VorwerkVittorio PengoJohannes ZenkVivek Pravin DaveJohn BerdahlVivien ChavanelleJohn BlakeVlad CiobotaruJohn C. DicksonVlad GorduzaJohn EllulVlad PorumbJohn FreanVlad TicaJohn GriniatsosVladareanu RaduJohn HarrisonVladimir I. GridnevJohn J. HealyVladimir L. GabaiJohn Ng'ombeVladimir LozanovskiJohn ReaganVladimir NikolenkoJohn S. MitchellVladimir PisarevJohn VirostkoVladimir SobolevJohn William BranchVladimir VaksJolanta Nawrocka-RutkowskaVladislav Vladimirovich TsukanovJolanta RzymowskaVladislava GusarJolanta SzymańskaVlasta FesslovaJolanta VaskelyteVolkhard HelmsJolanta-Justina VaskelyteVui King Vincent-ChongJon Salmanton-GarcíaWael Mohamed Shaher YafoozJonas BianchiWai San CheangJonas Nascimento CondeWakiro SatoJonathan BenzaquenWaleska DornasJonathan Frederik CarlsenWalid El-ShafaiJonathon Edward MohlWalter J. FlapperJong Hyo KimWalter L. StrohmaierJonghwa JangWalther BildJong-Hwa JangWan Aliaa Wan SulaimanJong-Hwan KimWang XiChiJong-Moon ChaeWasan KatipJongsub MoonWatshara ShoombuatongJonnathan Guadalupe Santillán-BenítezWattanapong KurdthongmeeJoo Beom EomWayne DimechJoon Hyuk ChoiWei ChengJoon Young ChoiWei LiJoost Van Der VorstWei NaiJordi Maitas-GuiuWei TengJordi TamaritWei WangJörg KleeffWei-Chieh LeeJörg RadermacherWei-Chih ChenJorge Galván-TejadaWei-Chun LinJorge H. LeitãoWeijia LiJorge Javier AlfonsoWeijin GuoJorge Murillo-GonzálezWei-Lun TsaiJorge N. R. MartinsWei-Min LiuJosch K. PaulingWeisi LiuJosé A. Noguera-VelascoWeiwei HanJose Alegre-MartínWeiwei ZhangJosé Antonio PintoWeiyan YinJosé António SimõesWells UtembeJosé Bañuls-RocaWen ShiJosé BlancoWencheng LiJosé ChiesWen-Chin LeeJosé De Jesús Rangel MagdalenoWen-Chun ChangJosé Francisco Rodríguez-VázquezWendy Scholten-PeetersJosé Higino CorreiaWenhao ShenJosé LarragaWenjie WangJosé Luis Del Castillo Pardo de VeraWen-Pin HuJosé Luis MansurWenwu LingJosé Luis Martín-ContyWenyi LuoJosé Luis Martín-VenturaWenyu WangJosé Luis Rodríguez GutiérrezWerner StreifJosé M. Palacios-JaraquemadaWesley KerrJose Manuel LopesWesley Luzetti FotoranJosé Manuel Marques Martins De AlmeidaWibke BayerJose Maria Pereira De GodoyWiesław LeonskiJosé Millet RoigWilfredo De Jesús-RojasJosé Raduan JaberWilliam JacotJose RodellarWilliam R. ReedJose SigutWilliam Y. RaynorJose-Antonio Mirón-CaneloWilly HauzerJosefa Gonzalez-SantosWing P. ChanJosé-María Sánchez-GonzálezWing-Keen YapJosep ArnabatWira WinardiJoseph ColomboWisit KaewputJoseph GeradtsWladimir Bocca Vieira De Rezende PintoJoseph LeachWładysław LasońJoseph NassifWladyslaw WeglarzJoseph PikkelWlodzimierz BaranowskiJosette RaymondWłodzimierz SkorupskiJosip A. BorovacWojciech CytawaJosip DelmisWojciech KiebzakJosko BozicWojciech KustrzyckiJoyce O'SheaWojciech PolomJózef W. KobosWojciech WitkiewiczJózsef TollárWolfgang BoettcherJuan A. Fafián-LaboraWolfgang FreysingerJuan Antonio Martínez-TadeoWolfgang WuerfelJuan ArciniegaWolfram GerlichJuan AyalaWonkeun SongJuan Bautista De SanctisWonse ParkJuan Carlos Amor-DoradoWonwoong LeeJuan Carlos López-GarcíaWoo June ChoiJuan DuWoo Kyung JeongJuan Garduño-EspinosaWoo Sik YooJuan Ivan Nieto-HipolitoWoon-Man KungJuan J. CerrolazaWynand AlkemaJuan Luis AlcázarXavier Gallart-PalauJuan Luis Muñoz BellidoXavier Sánchez-SáezJuan M. MelchorXenophon ZabulisJuan Manuel Guzmán-FloresXia WeizhongJuan Manuel Muriel-SánchezXianchuang ZhengJuan MelchorXiangjiu DingJuan P. Amezquita-SanchezXiangping ChuJuan Pedro Martínez RamónXiangyang XuJuan Suárez-AnteloXiangyu ZhangJuan-Andrés Martín-GonzaloXianqi LiJude HemanthXiaodong GeJudit OláhXiaoguang LiuJudith Catella-ChatronXiaohai ZhouJudith Dominguez-CheritXiaohong ZhouJudy E. SternXiaojun GuanJuha P. VäyrynenXiaoliang ChenJuilee RegeXiaoming ZhengJui-Ting HsuXiaoxu JiJui-Yang LaiXiaoyan ChenJulia Furtner-SrajerXiaoyu ChenJulia VoglerXiaoyu MaJulian TaugnerXin CuiJulián ValdésXin LiJulian WebberXin MuJuliana YoradnovaXin ZhouJulie BolcaenXindong MaJulie GuignotXing ZhangJulie MagatXingshun QiJulie MassartXingxing HaoJulien AncelXinlei LiJulio CostaXinmai YangJulio Francisco Jiménez-BonillaXóchitl TrujilloJulio Plaza DíazXue WangJulius HöhneXue-Bo JinJuliusz HuberXuejuan JiangJun HarumaXueying WangJun Hui JeongXuqiao ChenJun MiyazakiXuyi YueJun RenYaewon KimJun WanYafeng MaJun Woo AhnYan QiangJunchao TongYana Roka-MoiiaJung Sun YooYancong QiaoJungHwan OhYancy FerrerJung-Soo PyoYang YouJunhao LiYang-Gun SuhJunichi ShimizuYannick Fabian DiehmJun-ichi TanumaYao-Ching HungJunichi TsuchiyaYaodong GuJun-Jen LiuYaoxiang LiJunji YamauchiYaser Daanial KhanJunn-Liang ChangYashima KazuoJunran ZhangYasmeen GeorgeJunxiang ChenYassine HimeurJunya KusumotoYasue MitsukuraJure KnezYasuhiko AbeJure UrbancicYasuhiro MochidaJürgen GermannYasuhiro TakahashiJürgen RödelYasuki HoriJürgen SchlegelYasunari MatsuzakaJussi Wiljami JylkkäYasuo TakeuchiJustin P. HardickYasushi KotaniJustus MarquetandYasushi ShibataJustyna KowalskaYasutaka FushimiJustyna Małyszek-TumidajewiczYasuyuki KobayashiJustyna PaprockaYaswanth KuthatiJustyna SikoraYe WuJyotir Moy ChatterjeeYe ZhangJyotsna ShahYeji HanKaan OrhanYeliz KaracaKadir SabanciYenddy N. CarreroKah Hui WongYen-Kung ChenKah Kheng GohYen-Lin LiuKai ChenYen-Ta HuangKai WangYeon Hyeon ChoeKai WilleYeong Chan LeeKai-Cheng WangYeou-Jiunn ChenKaikai XuYi HuangKaisa Leea SunelaYi Sheng LaiKaisu Rantakokko-JalavaYi-Cheng ChenKalaska BartlomiejYicheng ZhuKalpana RajaYiğit ÜncüKamalesh ChakravartyYi-Hsing ChenKamalika MojumdarYi-Hsuan HsiaoKamen DimitrovYiing Chiing YapKamil DimililerYi-Jang LeeKamil M. FramYilai ShuKamil NelkeYiming TangKamonwon IenghongYiming ZhangKamred Udham SinghYinan JiangKamyar KhoshnevisanYing LuoKang Hao CheongYing S. ZouKankoé SallahYinggan TangKannamannadiar JayaprakasanYing-Ren ChienKaoruko ShimizuYin-Kai ChaoKapil JoshiYinzhou YanKarel AllegaertYi-Qing YangKaren L. ReaderYi-Wen ChiuKaren LomondYi-Yuan LinKaren MolyneuxYoad YagilKarim HameschYoav ArnsonKarin PfisterYohan KerbageKarina A. Wierzbowska-DrabikYohann DabiKarine EvangelistaYoichi ShimizuKarl BechterYon-Cheong WongKarl-Heinrich LinkYong An ChungKarol KarasiewiczYongfen XuKarol SteckiewiczYonghao XuKarol SzylukYongji ZengKarolina ChilickaYongkui LiKarolina MarkietYong-Moon LeeKaroly GulyaYongwon ChoKarrar AbdulkareemYoon-Chul KimKarthick KanagarathinamYoon-Ji KimKarthik RamamurthyYoonJung LeeKartika Afrida FauziaYoon-Seok ChungKatarina Steding-EhrenborgYoo-Ri ChungKatarzyna BanachYorgos GoletsisKatarzyna Donskow-ŁysoniewskaYorihisa OritaKatarzyna HolcmanYorito HattoriKatarzyna KaczyńskaYoshiaki ZaizenKatarzyna KopczewskaYoshifumi IkedaKatarzyna Kościelska-KasprzakYoshifumi SaijoKatarzyna Mańka-MalaraYoshihiko YanoKatarzyna Mazur-MelewskaYoshihiro FukumotoKatarzyna Mizia-StecYoshihito ShimaKatarzyna NeubauerYoshikane YamauchiKatarzyna NowomiejskaYoshikazu Honda-OkuboKatarzyna SiewkoYoshiki SakaguchiKatarzyna WalczakYoshinori AkaoKate McBrideYoshiro HoraiKaterina KambouriYoshitaka NakayamaKaterina RoubalovaYoshitaka ShimotaiKatharina Esser-NobisYoshiyuki NakamuraKatheleen J. GardinerYoucef M. DerbalKatherine R. CalvoYouichi Youichi OhnoKathleen PishasYoung Eun YoonKathryn FrietzeYoung HongKatia LeiteYoung UhKatja EggermannYoung YiKatrin ScheinemannYoung-Ji ShiaoKatsuaki IeguchiYoun-Jee ChungKatsuhiko KatoYousif SubhiKatsuhiko MorimotoYousuke NakaiKatsuhiro ItoYu Chieh KoKatsuki TsuchiyamaYu Jin LimKatsunori MatsuedaYu KoyamaKatsunori TeranishiYu Kyung ChoKatsushi OkuyamaYu SunKatsushi TakebayashiYu TakahashiKatsutoshi SugimotoYu Ting TsaiKatsuya KitamuraYu Yuan ZhangKatsuyuki MiyabeYuan XueKaushik SridharYu-Chao ChangKawsar AhmedYu-Cheng ChouKay Choong SeeYu-Chiang WangKayvan ZainabadiYu-Ching WenKazimierz KuliczkowskiYue ShengKazuhiko HashimotoYue ZhaoKazuhiko ImaizumiYuedan WangKazuhiro OmuraYuehua LiKazuhiro P. IzawaYuemin ZhuKazuhito SuzukiYugo AshinoKazuho HondaYuhei MatsudaKazuki IdeYuhi FujimotoKazunori NagasakaYu-Hsuan Joni ShaoKazuo OhtsukaYuichi YoshiiKazuto TajiriYuichiro HatanoKazuya ShiratoYuichiro IkebuchiKazuyuki WatanabeYuichiro J. SuzukiKee Huong LaiYuichiro KomaKei Hang Katie ChanYuichiro YoneokaKeigo UchimuraYuji ShimizuKeiichi KubotaYujiao LuKeiichi TominagaYuki HirataKeiji KurodaYuki IshikawaKeisuke MiyazawaYuki KataokaKeita KaiYuki NakamuraKeith RochfortYukiko MatsudaKelechi NjokuYuko HaradaKen KuduraYuko OnoKenan W. D. SternYu-Kwan ChenKenichi HongoYulia DavydovaKenichi SudaYuman LiKenichi YamadaYumin Huang-LinkKen-Ichiro MatsumotoYun FengKenji HayashidaYunchen YangKenji TsujiYung-Chieh LinKenjiro YamamotoYung-Hui HuangKenneth A. AndreoniYung-Luen YuKenneth R. FosterYuning HuKentaro IwataYun-Ju ChenKentaro YoshiokaYun-jung YangKenya KusunoseYuntao HuKetan KotechaYuri G. GordienkoKeti VitanovaYuri KrakKeun-Yeong JeongYuri PisarevskyKevin C. ProudYurii KistenevKe-Vin ChangYuriy DavidyukKevin EboigbodinYuriy S. BronshteynKevin HackshawYushin TakemotoKhairun SaddamiYushu GeKhalad HasanYusuf OmosunKhaled KhedherYusuf PerwejKhalid MasoodYusuke HashimotoKhalid Omer AlfaroukYusuke InoueKhin ThaYusuke KatayamaKhosrow MottaghyYusuke SekinoKhurshid AlamYusuke ShimakawaKihwan ChoiYu-Sun ChangKijin KimYuting WangKilto ChongYu-Wei LiuKimmo VirtanevaYvonne LiKimon DivarisZachary R. McCawKinga Hińcza-NowakZahid UllahKinga Humińska-LisowskaZahra El-SchichKiran Kumar Solingapuram SaiZaid Abdi Alkareem AlyasseriKiran SandhuZaid YounesKishore BharathyZaiga Nora-KrukleKishore RajendranZakuan DerisKi-Sun LeeZala LuznikKiwamu MatsuokaZaneta Swiderska-ChadajKiyoshi AsadaZara SteinmeyerKiyoshi TakagiZargari Marandi RamtinKiyoshi YaoedaZehra Oya UygunerKiyoto ShigaZekiye AltunKlaudija ViškovićZekuan YuKlaus TenbrockZeljko HocenskiKlearchos PsychogiosZeljko SundovKo KoZenon MariakKohei OkuyamaZenon PogorelićKoichi OkumuraZeynep Alpay SavasanKoji AkedaZhan LiuKoji KomoriZhen QiuKoji MitaZhenchang WangKoji OtaniZheng XuKoji TanakaZheng ZhongKok Siong ChenZhiguo ZhangKok-Min SeowZhijun GaoKollarigowda RavichandranZhilin QuKollur Shiva PrasadZhiping LinKomal AroraZhiping LiuKondapa Naidu BobbaZhiyun XueKonrad AumannZhonghua SunKonrad HuppiZhongmin QiuKonrad Kamil HozyaszZia Ur RehmanKonstantin U. NikolaevZidian XieKonstantinos A. ZikopoulosZifei LiangKonstantinos DritsasZissis C. ChroneosKonstantinos MitsakakisZivile UseckaiteKonstantinos StamatiouŽivka DikaKonstantinos StergiosZoard KrasznaiKonstantinos TsaknakisZoe FlorouKornelia KreiserZograb MakiyanKornelia ZarębaZohaib JanKota HoshinoZohaib YousafKota YokoyamaZohar KeidarKotaro HaruharaZoltan RuzsaKoumantakis GeorgeZozan GulekenKourosh SheibaniZsolt BacsoKovy Arteaga-LiviasZsolt NemethKoya OzawaZsuzsanna BereczkyKresimir DolicZsuzsanna MihályKrishna Mohan SurapaneniZuan-Tao LinKrishna YadavZubair AhmadKristijan SkokZubair KhanKristina Hellén-HalmeZubbair MalikKristoffer Lindskov HansenZulqurnain SabirKristy SwiderskiZuzana KozovskaKrunoslav SvericZuzana TatarovaKrzysztof DowgierdZuzanna NowickaKrzysztof GorynskiZuzanna RzepkaKrzysztof JamroziakZyanya Reyes-CastilloKrzysztof LetachowiczZygmunt Warzecha


